# Celiac plexus radiosurgery for retroperitoneal pain in advanced cancer: a pre-specified secondary analysis of health-related quality of life in a phase II single-arm trial

**DOI:** 10.1016/j.eclinm.2026.103968

**Published:** 2026-05-07

**Authors:** Aisling S. Barry, Ronen Fluss, Laura A. Dawson, David Hausner, Michael Buckstein, Talia Golin, Laurence Freedman, Marcin Miszczyk, Dayssy DiazPardo, Artur Aguiar, Dror Limon, Raphael M. Pfeffer, Moaz Ben-Ayun, Liat Hammer, Ziv Symon, Ofir Morag, Gali Jacobson, Adam Dicker, Jerzy Wydmanski, Camilla Zimmermann, Yaacov R. Lawrence

**Affiliations:** a Cancer Research@UCC, School of Medicine and Health, University College Cork, Department of Radiation Oncology, Cork University Hospital, Ireland; b The Biostatistics and Biomathematics Unit, The Gertner Institute for Health Policy and Epidemiology, Sheba Medical Center, Ramat Gan, Israel; c Department of Radiation Oncology, Princess Margaret Hospital Cancer Centre, University of Toronto, Toronto, ON, Canada; d Department of Palliative Care, Sheba Medical Center, Ramat Gan, Israel; e Department of Radiation Oncology, Icahn School of Medicine at Mount Sinai, New York, NY, USA; f Institute of Oncology, Chaim Sheba Medical Center, Tel Aviv University, Tel Aviv, Israel; g Department of Urology, Comprehensive Cancer Center, Medical University of Vienna, Vienna, Austria; h Department of Radiation Oncology, The Ohio State University Wexner Medical Center, Columbus, OH, USA; i Department of Radiation Oncology, University of Minnesota, USA; j Department of Radiation Oncology, Instituto Português de Oncologia, Porto, Portugal; k Department of Radiation Oncology, Rabin Medical Center, Petah Tikvah, Israel; l Department of Radiation Oncology, Assuta Medical Center, Tel Aviv, Israel; m The Benjamin Davidai Department of Radiation Oncology, Sheba Medical Center, Tel HaShomer, 5265601, Israel; n Department of Radiation Oncology, Thomas Jefferson University, Philidelphia, PA, USA; o Department of Supportive Care, Princess Margaret Hospital Cancer Centre, University of Toronto, Toronto, ON, Canada; p The Gray School of Medicine, Gray Faculty of Medical and Health Sciences, Tel Aviv University, Tel Aviv, Israel; q Department of Radiation Oncology, Sidney Kimmel Medical College at Thomas Jefferson University, Philadelphia, PA, USA; r Collegium Medicum - Faculty of Medicine, WSB University, Dąbrowa Górnicza, Poland; s IIIrd Radiotherapy and Chemotherapy Department, Maria Skłodowska-Curie National Research Institute of Oncology, Gliwice, Poland

**Keywords:** Pancreas cancer, Radiotherapy, SRS, SBRT, Celiac plexus, Pain

## Abstract

**Background:**

Retroperitoneal pain syndrome seen in pancreatic cancer is a major clinical challenge. Celiac plexus radiosurgery, a new palliative technique for retroperitoneal pain syndrome, decreased pain levels in a phase II study. Here Health-Related Quality of Life (HRQOL) outcomes are reported.

**Methods:**

Evaluable patients, who were enrolled between January 2018 and December 2021, in an international single-arm Phase II ethics-approved study (NCT03323489), were included. The pre-specified secondary endpoint–change in HRQOL from baseline to 3- and 6-weeks post-treatment, was measured by the Functional Assessment of Cancer Therapy – Hepatobiliary (FACT-Hep) version 4 questionnaire. Mean change was deemed clinically significant (CS) if the lower bound of the 95% confidence interval was above the predefined minimal clinically important difference (MCID) for that outcome.

**Findings:**

Of 90 evaluable patients, 51 (57%) and 44 (49%) had HRQOL FACT-Hep scores available at 3- and 6-weeks, compared to baseline. The majority of patients had pancreatic cancer (90%) and mean daily intravenous morphine equivalent consumption was 35.9 mg (SD 67.6).

The mean increase of FACT-Hep total score from baseline was 9.3 points at 3-weeks (p = 0.0017) and 19.6 points at 6-weeks (p < 0.0001, 95% CI 13–26.3), representing a statistically significant (SS) 22% improvement from baseline, with CS demonstrated at 6-weeks. A sensitivity analysis, imputing no change from baseline in patients with missing data, demonstrated similar results. On multivariable analysis, lower baseline opioid use and baseline FACT-Hep scores were associated with an increased change in FACT-Hep from baseline to 3-weeks.

**Interpretation:**

Celiac plexus radiosurgery was associated with a SS improvement in HRQOL amongst patients with retroperitoneal pain at 3-weeks, and a SS and CS improvement at 6-weeks post intervention.

**Funding:**

This study received major financial support provided by Gateway for Cancer Research (G-17-100), additional support was provided from the Israel Cancer Association (20170128 and 20181272).

**Research in context**

**Evidence before this study**

Three in four patients with pancreatic cancer present with pain at diagnosis, and in approximately one third of patients this pain is difficult to control with oral analgesics. Celiac plexus radiosurgery is a new technique targeting the anatomical basis of neuropathic pancreatic cancer pain with a single fraction of very high dose radiation. In an international single-arm multi-institutional phase II trial, this intervention significantly decreased pain levels 3- and 6-weeks post treatment, with minimal side effects. This paper analyses health-related quality of life (HRQOL) outcomes in patients with advanced cancer associated pain who were enrolled on a previously published single arm international multi-institutional phase II study and received celiac plexus radiosurgery. Cancer related abdominal pain can adversely impact HRQOL, with pharmacotherapy and neurolysis the most commonly used pain-modifying therapies with varying success rates. Celiac plexus radiosurgery is a new technique targeting the anatomical basis of pancreatic cancer pain.

**Added value of this study**

HRQOL was a secondary outcome measured using the Functional Assessment of Cancer Therapy – Hepatobiliary (FACT-HEP) at baseline, 3 weeks and 6 weeks post celiac plexus radiosurgery. We found that celiac plexus radiosurgery was associated with a statistically significant improvement in HRQOL at 3 weeks, and a statistical and clinical improvement at 6 weeks.

**Implications of all the available evidence**

Few studies have studied the effect of pain relief interventions on HRQOL in pancreatic cancer. This study describes HRQOL outcomes following celiac plexus radiosurgery in relation to the prospective international phase II study for which the primary endpoint was pain response. Validation in a randomised phase III trial is required.

## Introduction

Three in four patients with pancreatic cancer present with pain at diagnosis,^1^ and in approximately one third of patients this pain is difficult to control with oral analgesics.^2^ The pathophysiology of pancreatic cancer pain is thought to be both neuropathic, due to cancer infiltration of the retroperitoneal celiac plexus located directly behind the pancreas, and nociceptive, due to cancer-mediated tissue invasion and inflammation.^3^^,^^4^ Cancer related symptoms adversely impact health-related quality of life (HRQOL).^5^ In patients with metastatic pancreatic cancer, baseline HRQOL scores are impaired^6^ and deteriorate further over time.^7^ Pharmacotherapy^8^ and neurolysis^1^ are the most commonly used pain-modifying therapies in this population; however reported success rates for these interventions are inconsistent,^9^^,^^10^ and their impact upon HRQOL rarely measured.^11^

Celiac plexus radiosurgery is a new technique targeting the anatomical basis of neuropathic pancreatic cancer pain with a single fraction of very high dose radiation. Given the complex pathophysiology of pancreatic cancer-associated pain,^12^ we hypothesized that directly interrupting pain signal transmission, rather than relying on indirect pain relief through tumour burden reduction, may offer a faster and more durable response. Additionally, in a pathomorphological study in pancreatic cancer patients by Ceyhan et al.^13^ severe pain was associated with greater nerve hypertrophy and neural density, furthermore it was associated with a poor prognosis.

In an international single-arm multi-institutional phase II trial, this intervention significantly decreased pain levels 3- and 6-weeks post treatment, with minimal side effects.^14^ Here we report HRQOL outcomes from this trial. This manuscript aims to: 1) evaluate how celiac plexus radiosurgery affects HRQOL, 2) prospectively evaluate intervention-related gastrointestinal toxicity using the hepatobiliary cancer subscale (as prespecified in the Protocol), and 3) analyze the relationship between pain relief and quality of life in pancreatic cancer patients, an important but understudied association.

## Methods

### Trial summary

Evaluable patients, who were enrolled between January 2018 and December 2021, in an international single-arm Phase II ethics-approved study (NCT03323489), were included.^15^ All participants provided written informed consent prior to enrolment and any study-related radiotherapy procedures were undertaken.

### Study population

Key eligibility criteria were patients with an average pain level ≥5/11 on the Brief Pain Inventory–Short Form (BPI), performance level ECOG 0–2, and anatomical involvement of the celiac axis. Due to the near universal presence of perineal invasion in pancreatic cancer, any pancreatic cancer patient was eligible in this regard. Non-pancreatic cancer patients were required to demonstrate gross anatomical involvement of the celiac axis on imaging. The protocol defined an ‘evaluable patient’ as a patient eligible for enrolment who received the therapy per protocol and was alive 3-weeks post-treatment; (2) had BPI average pain score of ≥4 at the assessment immediately before the first treatment; (3) did not have a reduction of BPI between screening and immediately before treatment of greater than 2 points. Sex and ethnicity were self-defined.

### Intervention

The intervention was a single radiosurgical fraction of 25 Gy delivered to the celiac plexus, as previously described.^15^^,^^14^ The celiac plexus target volume was an approximately 11 cm long retroperitoneal structure, with treatment of adjacent tumor tissue left to physician discretion. A ‘pain responder’ was defined as a subject with a ‘complete or partial (≥2 points) pain response’, based on the BPI ‘average pain’ 11-point subscale at 3 weeks.

### Outcome of interest

Change in HRQOL from baseline to three was a pre-specified secondary endpoint of the primary study,^14^ as measured by the Functional Assessment of Cancer Therapy – Hepatobiliary (FACT-Hep)^16^ version 4 questionnaire. This is a specific HRQOL questionnaire for patients with hepatobiliary cancers,^17^ with reported validity and reliability in patients with metastatic pancreas cancer.^17^^,^^18^ HRQOL was assessed at three timepoints – baseline (before treatment), 3 weeks, and 6 weeks post-treatment. Changes in FACT-Hep scores from baseline to 3- and 6-weeks were assessed in patients with both baseline and 3- and 6-week scores. Difference scores were calculated by subtracting the baseline from the 3- and 6-week scores.

The FACT-Hep tool is a 45-item self-administered health-related questionnaire that assesses generic HRQOL issues according to five sub-domains – physical well-being (PWB), social/family well-being (SWB), emotional well-being (EWB), functional well-being (FWB), the hepatobiliary cancer subscale (HCS)^16^ and summary scores–Trial Outcome Index (TOI) which incorporates PWB + FWB + HCS, Total FACT-General [FACT-G] (PWB + FWB + SWB + EWB) and FACT-Hep total score (incorporates all domains).^19^ A higher score indicates better HRQOL.

The minimal clinically important difference (MCID) measures the smallest change in a patient's quality of life that is considered worthwhile.^20^ Using MCID estimates determined by Steel et al.,^21^ a FACT-Hep score of 8.5 points, FACT-G score of 7, and TOI score of 8 were used to assess improvement or deterioration. MCIDs for FACT-G subscales (PWB, SWB, EWB, FWB) were defined as a 3-point change, and for HCS a 5.5-point change.^21^

### Statistical analysis

Baseline characteristics of the patient population and each domain and subscale were summarised using means and standard deviations (SD) for normally distributed continuous variables, and medians with interquartile ranges (IQR: Q1 – Q3) for non-normally distributed continuous variables. Normality of distribution was assessed using the Shapiro–Wilk test for normality and visually based on histograms/QQ plots of each continuous variable (Supplementary Table S3). In the case of non-normally distributed variables, non-parametric tests, such as Wilcoxon ranked sum test, were used for analysis. Categorical variables were summarized using frequencies and proportions, and relationships examined using chi-square tests or Fisher exact test (Supplementary Table S4). Prior to analysis, assumptions of chi-square tests were evaluated ensuring observations were independent and expected cell counts in 80% were ≥5 (Fisher exact tests were used instead if expected cell counts were <5). Continuous variables were compared using inter- and intra-group comparisons with Student's t-tests and Wilcoxon ranked sum where appropriate.

Linear regression assessed the relationship between mean change score (baseline to 3-weeks) and clinically relevant predictor variables, including opioid use. Proportions were used to summarise MCID and the number of patients who had a change in any direction at 3-weeks post-intervention. 95% Confidence Intervals (CI) for each subscale were reported in relation to their predefined MCIDs; as per protocol, the mean change was deemed clinically significant only if the lower bound of the 95% CI was above the predefined MCID for that outcome.^15^ A subgroup analysis assessed differences in baseline characteristics and HRQOL change scores between pain responders and non-responders using Student's t-test, Wilcoxon ranked sum, and chi-square tests as appropriate.

A two-tailed p-value of ≤0.05 was considered statistically significant (SS). SAS/STAT software™ version 9.4 (SAS Institute, Cary, NC, USA) was used for data analysis. The Bonferroni correction method for multiple comparisons was incorporated into the FACT-Hep and FACT-G multi scale analysis, with a corrected p-value of <0.006 used.

### Sample size calculation

As per the previously published protocol^15^ the sample size was determined by justifying that CP SRS would be successful in at least 40% of enrolled patients. Assuming a true response rate of 60% a trial of 90 patients will have a 96.5% chance of demonstrating a one-sided statistical significance level of 2.5% with no dropout.

### Handling of missing data

HRQOL data were reviewed for missingness. Based on the FACT-Hep scoring manual version 4, subscales were calculated if more than 50% of items were answered, with a total score calculated when all components of the subscales had valid scores. Where HRQOL outcomes at 3-weeks were missing, they were dropped from the primary analysis. We assumed ‘missing not at random’ as a sensitivity analysis and performed a protocol-defined sensitivity analysis by imputing zero change from baseline. Additional post-hoc sensitivity analyses were performed to assess robustness of this primary analysis. Firstly, the assumption of a clinically meaningful HRQOL worsening, which the protocol defined as MCID value of −8.5 points was imputed for all missing 3-week values. Secondly, pain interference (from BPI) was used as a predictor of change in FACT-Hep at 3-weeks. We, however, considered it clinically implausible that all missing patients experienced the maximum possible decline, therefore we conservatively assumed that all patients with missing 3-week data, and could not model their HRQOL based upon pain-interference, experienced a deterioration equivalent to the MCID. This represents a conservative but clinically grounded worst-case assumption. A sample correlation was performed between change in pain interference score and FACT-Hep score at 3-weeks. For patients (N = 21, 41%) with a change in pain interference and missing FACT-HEP at 3-weeks, the value was imputed by fitting a linear regression model between pain interference and FACT-HEP change. Repeated imputations were performed and the estimated mean FACT-HEP change and its standard error were pooled using Rubin's rule.

### Ethical approval

The trial was approved by the Sheba Medical Center's ethics committee (SMC-17-4292), and by each subsite's ethics committee.

### Role of the funding source

The funders of the study had no role in study design, data collection, data analysis, data interpretation, or writing of the report.

## Results

### Population and pain response summary

Between 2018 and 2022, 149 patients were enrolled, of whom 125 received treatment and 90 were evaluable for analysis. Patient characteristics, treatment details and the primary endpoint of pain score change have been published.^14^ Briefly, 53% of patients had at least a partial pain response at 3-weeks, opioid consumption was stable at 3-weeks post-treatment and decreased by 30% at 6-weeks.

### Health-related quality of life – FACT-Hep results

Of the 90 evaluable patients, 51 (57%) with HRQOL FACT-Hep scores available at baseline, and 3-weeks, and 44 (49%) with baseline and 6-weeks scores, were included in HRQOL analyses. Compared to the 39 (43%) evaluable patients with missing HRQOL outcomes the two groups had no significant differences in most baseline characteristics (Table 1). Mean baseline FACT-HEP total scores for the included study's population (Number of patients [N] = 51), vs non-study population (N = 39), were 90.5 vs 84.7, respectively (p = 0.16). However, median survival was significantly longer in patients with 3-week HRQOL scores available, at 149 (IQR 105–267) days, compared to 79 (IQR 49–141) days (p < 0.0002).

**Table 1:** Baseline Characteristics of evaluable patients, comparing the analysable vs the non-analysable population – based upon the availability of HRQOL outcome data.

Variable	Analysable population^a^ N = 51 (57%)	Non-analysable population^b^ N = 39 (43%)	p-value
Gender			
Male	23 (45.1)	17 (43.6)	
Female	28 (54.9)	22 (56.4)	0.88
Primary diagnosis			
Pancreas cancer	46 (90)	37 (95)	
Other	5 (10)	2 (5)	0.41
Baseline ECOG			
0	3 (6)	1 (2.5)	
1	31 (61)	28 (72)	
2	17 (33)	10 (24.5)	0.59
Prior lines of systemic therapy			
1	27 (54)	19 (50)	
>1	23 (46)	19 (50)	
Unknown	1	1	0.71
CP invasion due to			
Primary cancer	32 (78)	27 (75)	
Metastatic disease	5 (12)	3 (8)	
Local recurrent disease	4 (10)	6 (17)	
Unknown	10	3	0.61
Overall survival			
(Med, IQR) days	149 (105–267)	79 (49–141)	
Missing (N)	0	0	<0.0002
Baseline pain score			
(Med, range)	6 (4–10)	6.5 (5–10)	
Missing (N)	0	0	0.11
Baseline Opioid			
(Med, IQR) intravenous morphine equivalent, mg	35.9 (16.8–76.7)	24.3 (7.98–65.5)	
Missing (N)	1	1	0.19
Baseline FACT-HEP total score (Mean, SD)	90.5 (21.8)	84.7 (15.7)^c^	
Missing (N)	0	15	0.16

Acronyms: HRQOL – Health-related quality of life; N – number of patients; ECOG – Eastern Cooperative Oncology Group performance status; CP – celiac plexus; Med – median; IQR – interquartile range; SD – standard deviation; FACT-HEP – Functional Assessment of Cancer Therapy – Hepatobiliary; N – number of patients.

a Analyzable population – both baseline and 3-week HRQOL scores available.

b Non-analyzable population – lacking baseline or 3-week scores.

c Includes 24 patients with baseline HRQOL scores only.

The mean FACT-Hep total scores, at baseline, 3- and 6-weeks were 90.5 (SD 21.8), 99.8 (SD 26.1) and 109.7 (SD 22.5) (Table 2), a numerical improvement over time. The average FACT-Hep total score increased from baseline by 9.3 (95% CI 3.6–14.9) points at 3 weeks (p = 0.0017) and 19.6 (95% CI 13–26.3) points at 6 weeks (p < 0.0001, 95% CI 13–26.3) (Table 2). Although average changes at both time points were statistically significant and demonstrated a point estimate of >8.5, the 95% CI at 3-weeks included the MCID, insufficient to confirm clinical significance based upon the protocol definition. However, there was protocol defined clinical significance at 6-weeks (Table 3).

**Table 2:** Summary of FACT-HEP, FACT-G and subscale mean scores at baseline, 3- and 6-weeks post celiac plexus radiosurgery.

Evaluable population N = 51	Baseline	3-weeks	6-weeks	3-week change score^a^	6-weeks change score^b^	p-value 3-week^c^	p-value 6-week^d^
FACT-HEP						0.0017^e^	<0.0001^e^
Mean (SD)	90.5 (21.8)	99.8 (26.1)	109.7 (22.5)	9.3 (20)	19.6 (20.7)		
95% CI				3.6–14.9	13–26.3		
FACT-G						0.0043^e^	<0.0001^e^
Mean (SD)	60.7 (16.1)	66.6 (17.8)	72.9 (16.9)	5.9 (14.2)	11.9 (14.8)		
95% CI				1.9–9.9	7.2–16.2		
HCS						0.011	<0.0001^e^
Mean (SD)	29.8 (8.9)	33.2 (10.1)	37.1 (8.2)	3.3 (8.9)	7.1 (9.7)		
95% CI				0.8–5.8	4–10.2		
TOI						0.0010^e^	<0.0001^e^
Mean (SD)	52.1 (17)	60.1 (20.9)	68.5 (16.7)	7.5 (16.1)	16.1 (17.6)		
95% CI				3.3–12.4	10.5–21.7		
PWB - Physical Well-Being						0.0001^e^	<0.0001^e^
Mean (SD)	3.1 (5.3)	6.3 (6.6)	9.3 (5.2)	3.2 (5.5)	5.8 (5.9)		
95% CI				1.7–4.8	3.9–7.6		
FWB - Functional Well-Being						0.19	0.020
Mean (SD)	19.2 (6.6)	20.5 (6.9)	22.3 (6.4)	1.3 (6.9)	2.7 (7.2)		
95% CI				−0.6 to 3.2	0.4–5		
EWB - Emotional Well-Being						0.030	0.0028^e^
Mean (SD)	9.2 (5.9)	10.7 (5.5)	11.3 (6.2)	1.5 (4.7)	2.5 (4.9)		
95% CI				0.1–2.7	0.9–4		
SWB - Social Well-Being						0.96	0.13
Mean (SD)	29.1 (4.3)	29.1 (4.4)	30 (4.2)	−0.03 (4.9)	0.9 (3.9)		
95% CI				−1.4 to 1.3	−0.2 to 2.1		

For all these measures an increased score indicates improved HRQOL. Accepted minimal clinically important difference (MCID) for these scores are: FACT-Hep - 8.5 points, FACT-G–7 points, for FACT-G subscales (PWB, SWB, EWB, FWB)–3 points, TOI–8 points and HCS–5.5 points.

Acronyms: SD – standard deviation; 95% CI – 95% confidence interval; FACT-HEP – Functional Assessment of Cancer Therapy – Hepatobiliary; FACT-G - FACT-HEP – Functional Assessment of Cancer Therapy – General; HCS – hepatobiliary cancer subscale; TOI – Trial Outcome Index.

a 3-week change score = mean of 3-week score minus baseline score.

b 6-week change score = mean of 6-week score minus baseline score.

c p-value = 3-week vs baseline.

d p-value = 6-week vs baseline.

e Statistically significant on Bonferroni correction p = 0.006.

**Table 3:** Proportion of patients with minimal clinically important difference (MCID) changes in FACT-Hep at 3- and 6-weeks post celiac plexus radiosurgery.

	FACT-Hep		
	Improved >8.5 N (%)	Stable −8.5≤ to ≤+8.5 N (%)	Worsened < −8.5 N (%)
3-weeks overall N = 51	27 (52.9)	13 (25.5)	11 (21.6)
Pain responders N = 29	19 (65.6)	5 (17.3)	5 (17.3)
Non-Responders N = 22	8 (36.4)	8 (36.4)	6 (27.2)
6-weeks overall N = 40	31 (77.5)	5 (12.5)	4 (10)
Pain responders N = 24	21 (87.5)	1 (4.1)	2 (8.4)
Non-Responders N = 16	10 (62.5)	4 (25)	2 (12.5)

‘Pain responders’ and ‘Non-responders’ were protocol defined based upon the pain response at three weeks post treatment.

Acronyms: MCID – Minimally Clinically Important Difference; FACT-HEP - Functional Assessment of Cancer Therapy – Hepatobiliary; SRS – Stereotactic Radiosurgery.

We sought to understand which patients demonstrated the most improvement in FACT-Hep score from baseline to 3 weeks. Using univariate linear regression of the clinically relevant predictor variables (gender, baseline performance status, baseline FACT-Hep score, baseline opioid use, and baseline BPI score), only baseline opioid use (p = 0.050) was significant. On multivariable analysis, both lower baseline opioid use (p = 0.026) and worse baseline FACT-HEP scores (p = 0.041) were significant predictors of a greater mean change in scores (Table 4).

**Table 4:** Univariable and Multivariable Regression Analysis of FACT-HEP change score at 3 weeks post celiac plexus radiosurgery.

Variable	Univariable analysis			Multivariable analysis		
	Regression coefficient	Standard error	p-value	Regression coefficient	Standard error	p-value
Baseline FACT-HEP score N = 51	−0.26	0.14	0.071	−0.28	0.13	0.041
Gender	1.73	6.27	0.78			
Baseline performance status N = 51	3.6	4.98	0.47			
Baseline Opioid consumption N = 51	−0.09	0.04	0.050	−0.09	0.04	0.026
Baseline BPI score N = 51	0.48	1.9	0.80			

Acronyms: FACT-HEP – Functional Assessment of Cancer Therapy – Hepatobiliary; BPI – brief pain inventory; N – number of patients.

When a sensitivity analysis was applied by imputing missing 3- and 6-week FACT-Hep scores as *no change* in all 75 patients for whom baseline scores were available, mean FACT-Hep was 88.9 (SD 20.2) at baseline, 95.2 (SD 24.2) at 3-weeks, and 98.5 (SD 24.1) at 6-weeks. Similar to non-imputed results, the average improvements (3-weeks: 6.3, 95% CI 2.4–10.1; 6-weeks: 9.9, 95% CI 9.5–14.3) were statistically significant at both timepoints (3 weeks: p = 0.0020; 6-weeks: p < 0.0001), and clinically significant at 6-weeks. On further post-hoc sensitivity analysis, firstly assuming a clinically meaningful deterioration of −8.5 points at 3-weeks for all missing values, mean 3-week FACT-Hep was 92.5 (SD 25.7), a non-SS improvement from baseline (p = 0.076) was found. Secondly, imputing missing HRQOL scores using pain interference scores (correlation coefficient −0.42) and repeated 100 imputations using estimated intercept (3.27), slope (−2.7) and the error standard deviation (18.4), an non-SS improvement (4.31, p-value = 0.056) was found.

**Fig. 1:**
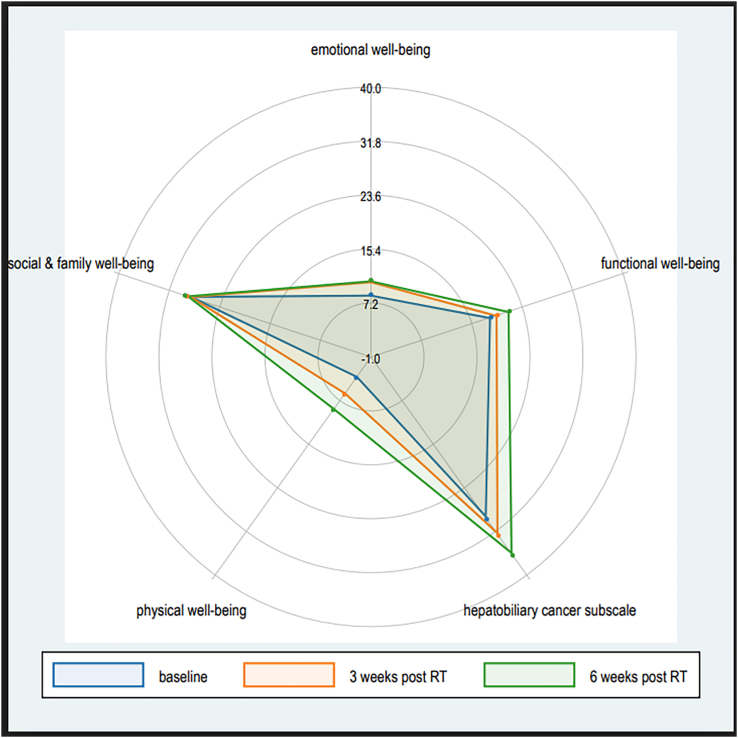
**A Spider Chart of FACT-Hep subscales,** Emotional, Social, Physical and Functional Well-Being and the Hepatobiliary Cancer, demonstrating mean change from baseline. The maximal score for all the subscales is 28, with the exception of ‘emotional wellbeing’ – maximal score 24, and the hepatobiliary cancer subscale – maximal score 72. Acronyms: RT – radiotherapy.

The FACT-G total scores had a statistically significant average increase at 3- and 6-weeks (5.9, p = 0.0043 and 11.9, p < 0.0001, respectively) and clinically significant at 6-weeks (11.9, 95% CI 7.2–16.2). In the FACT-G subscales, PWB demonstrated a SS improvement from baseline at 3- and 6-weeks, also demonstrating clinical significance at 6-weeks. EWB was SS, but not clinically significantly, improved at 6-weeks. The HCS, a protocol predefined subscale measure of assessing if QOL changed due to treatment related adverse events, did not show a statistically significant nor clinically significant, improvement at 3- and 6-weeks. Of note, adverse-events have previously been reported,^14^ in summary 11 grade 3 or worse adverse events were probably (n = 2) or possibly (n = 7) attributed to treatment. Although the adverse even data were difficult to interpret as previously discussed,^14^ they did not appear to indicate significant treatment-related toxicity beyond mild nausea and fatigue immediately following treatment. The TOI score, which assesses the impact of the trial intervention longitudinally by combining physical and functional well-being with HCS, demonstrated a statistically significant improvement at both timepoints, with clinical significance demonstrated at 6-weeks (Table 2, Fig. 1).

### Results for pain responders vs non-responders

Forty-eight patients (53% of those evaluable) were classified as pain responders and 42 as non-responders (NR). Twenty-nine (60%) responders and 22 (52%) NR had baseline and 3-week FACT-Hep scores available for analysis. Baseline characteristics were similar between groups; however, the baseline pain score was significantly higher in the responder group vs NR (p = 0.035) (Supplemental Table S1).

Baseline FACT-Hep scores were similar between the pain responder and NR groups (Supplemental Table S2). On intra-group comparisons, at 3-weeks, pain responders had a statistically significant improvement from baseline in FACT-HEP (p = 0.0004) which was not clinically significant, however, at 6-weeks, both statistically and clinically significant improvements were seen in FACT-HEP (p < 0.0001). In the NR group, a statistically significant, but not clinically significant, improvement from baseline was seen in FACT-HEP (p = 0.0091) at 6-weeks. On inter-group comparisons (pain responder vs NR), there was a statistically significant higher improvement in the 3-week score in the pain responder group compared to NR (14.8 vs 2.0 p = 0.031), with no significant difference at 6-weeks (22.8 vs 14.8, p = 0.11).

### Relationship between change in BPI and FACT-HEP at 3-weeks

The average BPI change score from baseline to 3-weeks was −2.7 (SD 2.6). BPI was negatively correlated (−0.34, p = 0.010) with change in FACT-HEP at 3-weeks, suggesting that HRQOL increased as pain decreased.

## Discussion

This study describes HRQOL outcomes following celiac plexus radiosurgery, based upon a prospective international phase II study for which the primary endpoint was pain response. A statistically significant improvement in FACT-Hep total score was demonstrated at 3- and 6-weeks, with clinical significance at 6-weeks. Furthermore, there was a statistically significant improvement in PWB and TOI subscales at 3- and 6-weeks and in EWB and HCS at 6-weeks, with a clinically significant change in TOI and PWB subscales at 6-weeks.

Compared with the general population, patients with pancreatic cancer have profoundly impaired HRQOL^22^ that deteriorates with time.^7^ Determinants of poor HRQOL amongst these patients include advanced disease stage, time from diagnosis, and symptom burden, including pain level. Few studies have studied the effect of pain relief interventions on HRQOL in pancreatic cancer.^9^^,^^11^^,^^23^^,^^24^ In a randomized trial conducted amongst 109 patients with inoperable abdominal or pelvic cancer, 38 of whom had pancreatic cancer, early neurolytic sympathectomy led to better pain control and comparatively better HRQOL (measured with the European Organisation for Research and Treatment of Cancer [EORTC] Quality of Life Questionnaire-Core 30 [QLQ-C30]),^23^ although it decreased in both arms compared to baseline over time. Other studies examined the effect of radiation therapy targeting the tumor on pain and HRQOL. A single-arm prospective study investigated the effect of gemcitabine followed by stereotactic body radiotherapy (SBRT) in 49 patients with locally advanced non-metastatic pancreatic cancer (targeting the tumour, not the celiac plexus)^25^; there was improvement in pain, with stable HRQOL at 4- and 16-weeks post RT. In a study that prospectively assessed the use of SBRT in 31 patients with locally advanced and metastatic pancreatic cancer (targeting the tumour), there was improvement in pain at one-month post-treatment, accompanied by improved HRQOOL (FACT-Hep total score at 1-month compared to 1-week pre-SBRT, 89.61 ± 6.59 vs 76.29 ± 6.28); however, pain returned to baseline by 3 months.^26^ Lastly, the PAINPANC^24^ phase II study assessed the use of short-course palliative radiotherapy (24 Gray in 3 fractions [days 1, 8 and 15]) to the primary pancreatic tumour and surrounding fatty tissues (the celiac plexus was not directly targeted) in 29 patients with refractory cancer related pain of ≥5 (0–10 BPI scale). This study demonstrated a statistically significant improvement in pain at 3-weeks; in addition, there was a statistically and clinically relevant improvement in Global QOL, using the EORTC Quality of Life Questionnaire-C15-PAL (quality of life in palliative cancer care patients questionnaire) at 5-weeks post-treatment.

On subgroup analysis of pain responders vs non-responders (NR) at 3- and 6-weeks post-treatment, both subgroups significantly improved in FACT-HEP scores from baseline, with responders experiencing a greater improvement than NR at 3-weeks but not 6-weeks. Interestingly, in the group of non-responders 36% and 62% of patients had improved HRQOL (>8.5 point positive change) at 3- and 6- weeks, respectively. These findings may reflect inaccuracies in the outcomes reported using the BPI-SF (for instance, due to subjects being inconsistent with scoring pain intensity scores, a ‘response shift’) and/or that the maximal pain response to celiac plexus radiosurgery occurs after 3-weeks. This may reflect improvements in other cancer-related symptoms such as fatigue, nausea, or psychological distress, though future research should investigate these mechanisms more systematically.

Aside from radiotherapy, current standard options for managing celiac plexus-related cancer pain consist of celiac plexus block (CPB) and/or pain medication, with CPB more often considered when pain is refractory to opioid analgesics. Although the terms are frequently used interchangeably, celiac plexus block involves local anaesthetic injection; in contrast, celiac plexus neurolysis involves injection of a neurotoxic agent such as phenol or ethanol. There are conflicting and limited data regarding the impact of CPB on the HRQOL of patients with pancreatic cancer. In a single-arm prospective study of 16 patients,^11^ CPB improved pain and QOL (Short form-36 questionnaire) at 35 days post-procedure. In a randomized double blind trial of 100 patients with unresectable pancreatic cancer experiencing pain, neurolytic CPB significantly improved pain compared to sham injection, albeit with small differences in pain intensity and increased opioid consumption in both arms.^9^ HRQOL improved in both groups at week one, but thereafter, both groups experienced a similar decline in HRQOL. The authors hypothesized that the small difference in pain scores between groups was insufficient to affect HRQOL estimated by the FACT-Pancreatic Cancer short form; it is also possible that the increasing doses of opioids consumed by subjects during the study impacted HRQOL. In a similar randomized controlled trial comparing endoscopic ultrasound-guided celiac plexus neurolysis to conventional pain management, pain relief improved in the intervention arm at 1- and 3- months post-procedure. Still, there was no difference in the mean percentage change in the HRQOL (using the Digestive Disease Questionnaire-15) between groups.^27^ These findings contrast our study, which was associated with improvement in reported pain and HRQOL post intervention, with stable or decreasing opioid consumption.

Our study has a number of strengths: (1) the study uniquely targeted the celiac plexus with high-dose radiosurgery, with an option of irradiating the adjacent tumour to a moderately lower dose.^14^ Of note, patients in the study had very advanced disease. This is reflected in the baseline demographics: at accrual, the median time from diagnosis was over 300 days, patients were taking large doses of narcotic analgesics, and the majority had received more than one line of systemic therapy. This is also reflected in the baseline HRQOL FACT-Hep scores, which were much worse (mean 90.5) than in many pancreatic cancer trials (range, 138.6–141.3).^28^^,^^29^ (2) In the current study, the predefined criteria for a clinically significant outcome were strict, incorporating statistical anchor-based methods using a confidence interval to determine significance. In the above-described studies, a clinically meaningful outcome was defined as a change from baseline in points only, with no requirement for the 95% confidence interval. If this study had defined clinical significance using point change only, it would also have demonstrated clinical significance in the primary outcome of FACT-HEP at 3- weeks. The 95% CI provides further statistical certainty that our trial demonstrated clinical significance at 6-weeks. (3) Additionally, the TOI is often used as an endpoint in clinical trials, due to its responsiveness to change in physical and functional scales; this is particularly relevant for trials of physical interventions, which are less likely to lead to a rapid change in social and emotional well-being subscales.^19^ In our study, the TOI was significantly improved at both time-points, with a clinically significant change at 6-weeks, further supporting the longitudinal positive impact of celiac plexus radiosurgery on patients’ HRQOL.

This preplanned secondary analysis is not without limitations. The principal limitation of this study is its single-arm design; the absence of a randomised control group precludes causal inference regarding the effect of celiac plexus radiosurgery on HRQOL. Additionally, the study was not powered to detect clinically meaningful HRQOL changes (a secondary endpoint), which may affect the precision of the estimates and the ability to demonstrate clinical significance at 3 weeks. Causal inference from factors such as baseline performance status, prior use of systemic therapy, opioid consumption, were considered in MVA, however inference from behavioural biases such as placebo/Hawthorne effects may still occur due to the lack of a control group. There is the potential for loss to follow-up bias, non-analysable patients had a shorter prognosis. However, we have attempted to account for this by imputation with a protocol defined sensitivity analysis, which yielded similar results. The predefined sensitivity analysis assumed no change from time zero, or last-observation carry forward, which may not be considered ‘worst case scenario’. However, additional sensitivity analysis performed demonstrated similar results showing an improvement from baseline, although not SS. Questionnaires were only completed at 3- and 6-weeks post-treatment and it may be possible that a longer follow-up would have revealed further improvement beyond that time. HRQOL data cannot account for a patient's response shift; patients may adjust their QOL priorities as disease progresses. Furthermore, floor and ceiling effects may impact the ability to detect change in this population, where the baseline HRQOL scores are low and minor effects may result in reduced scale discrimination to observe change.

Celiac plexus radiosurgery is associated with a statistically significant improvement in HRQOL amongst patients with retroperitoneal pain syndrome at 3- weeks post intervention, with a statistically and clinically significant improvement at 6- weeks. Validation in a randomised phase III trial is required.

## Contributors

All authors have read and approved the final version of this manuscript. ASB, YRL and RF, accessed and verified the data.

Conceptualisation: RF, CZ, YRL, LAD, DH, MB, TG, LF, AA.

Data curation: ASB, RF, YRL, LAD, DH, MB, TG, LF, MM, AA, CZ.

Formal Analysis and verification of underlying data: ASB, RF, YRL, LAD, DH, MB, TG, LF, MM, AA.

Methodology: ASB, CZ, RF, YRL, DH, MB, TG, LF, DD, DL, RP, MB, OM, GJ, AD, JW.

Project administration: ASB, RF, YRL, MB, TG, LF, MM, DD.

Resources, software, supervision: ASB, RF, YRL, LAD, DH, MB, TG, LF, MM, DD, CZ.

Validation and verification of underlying data: AS, RF, YRL.

Visualisation: ASB, RF, YRL, LAD, DH, MB, TG, LF, MM, DL, DD, RP, MB, LH, ZS, OM, GJ, AD, JW, CZ.

Writing–original: ASB, RF, LAD, DH, MB, TG, LF, MM, DL, DD, RP, MB, LH, ZS, OM, GJ, AD, JW, CZ, YRL.

Writing – review, editing and final draft: ASB, RF, LAD, DH, MB, TG, LF, MM, DD, AA, DL, RP, MB, LH, ZS, OM, GJ, AD, JW, CZ, YRL.

## Data sharing statement

Specific data requests by academic researchers who provide a methodologically sound proposal will be considered by the principal investigator (YRL) for 5 years after publication. A data sharing plan was not included in the trial protocol and hence data sharing will be conditional upon receiving the approval of the Institutional Review Board of the Sheba Medical Center. A data access agreement will be required for access.

## Declaration of interests

YRL: received research funding from Karyopharm Therapeutics, Checkmate Pharmaceuticals (purchased by Regeneron Pharmaceuticals), and Bristol-Myers Squibb; YRL received Honoria/consultancy fees from Bristol-Myers Squibb, Clinigen Group, Roche Genetech, Zola Therapeutics Inc., Radformation Stock ownership: Protean Biodiagnostics Inc.

LAD: Grant funding to institution (Elekta, Varian, Merck); Consulting (Astro Zeneca, Elekta).

APD: Dr. Dicker is an employee of Thomas Jefferson University/Jefferson Health Additional support from: National Cancer Institute, NRG Oncology, Prostate Cancer Foundation Challenge Grant Current Advisory activities -American Association of Cancer Research, Janssen, Oncohost, CVS.

The following co-authors declare no conflicts of interest or disclosures: ASB, RF, DH, MB, TG, LF, MM, DD, AA, DL, RP, MBA, ZS, OM, TM, GJ, LH, JW, CZ.

## Appendix A Supplementary data

Supplementary Table 4

CPSRS.Protocol
