# Supplementary material for: Celiac plexus radiosurgery for retroperitoneal pain in advanced cancer: a pre-specified secondary analysis of health-related quality of life in a phase II single-arm trial
Source: eClinicalMedicine. 2026 May 7;95:103968. doi: 10.1016/j.eclinm.2026.103968 (PMC13186082; doi:10.1016/j.eclinm.2026.103968)
Supplement: Supplementary Tables 1 and 2 [file mmc1.docx]

**Supplementary Table 1. Baseline Characteristics of patients who reported HRQOL, pain responders versus non-responders at 3-weeks post celiac plexus radiosurgery**.

*Pain response is based upon the change in pain score from baseline to 3 weeks.

| Variable | Pain Responder  N=29 (%) | Non-Responder  N=22 (%) | p-value |
| --- | --- | --- | --- |
| Gender  Male  Female | 13 (44.8)  16 (55.2) | 10 (45.5)  12 (54.5) | 0.96 |
| Primary diagnosis  Pancreas cancer  Other | 26 (89.6)  3 (10.4) | 20 (90.9)  2 (9.1) | 0.35 |
| Baseline ECOG  0  1  2 | 2 (6.9)  16 (55.2)  11 (37.9) | 1 (4.5)  15 (68.2)  6 (27.3) | 0.071 |
| Prior lines of systemic therapy  1  >1  unknown | 16 (57.1)  12 (42.9)  1 | 11 (50)  11 (50)  0 | 0.61 |
| CP invasion due to  Primary cancer  Metastatic disease  Local recurrent disease  unknown | 16 (76.2)  3 (14.3)  2 (9.5)  8 | 16 (80)  2 (10)  2 (10)  2 | 0.13 |
| Survival (Med, IQR) days | 174 (125 – 293) | 127 (100 – 178) | 0.089 |
| Baseline pain score (Med, range) | 6 (5-10) | 5.5 (4 – 10) | **0.035** |
| Baseline Opioid (Median, IQR) | 31.8 (14 – 53.3) | 39.8 (20 – 120.2) | 0.23 |

*Acronyms – HRQOL – Health-related quality of life; N - number of patients; ECOG – Eastern Cooperative Oncology Group; CP – celiac plexus; Med – median; IQR – interquartile range; SD – standard deviation; FACT-HEP - Functional Assessment of Cancer Therapy – Hepatobiliary*

**Supplementary Table 2. HRQOL scores of pain responders versus non-responders at 3-weeks and 6-weeks post celiac plexus radiosurgery.** Pain response was classified based upon the 3-week change in pain intensity.

|  | Pain Resp | | NR | Pain Resp | | NR | Pain Resp | | NR | | Pain Resp | | NR | | Pain Resp | | NR |
| --- | --- | --- | --- | --- | --- | --- | --- | --- | --- | --- | --- | --- | --- | --- | --- | --- | --- |
|  | Baseline | | | 3-weeks | | | 6-weeks | | | | 3-week change score | | | | 6-weeks change score | | |
| FACT-HEP  Mean (SD)  95%CI | 91.1 (21.4) | | 89.9 (22.8) | 105.9 (23.4) | | 91.9 (27.8) | 114.7 (21.4) | | 102.2 (22.6) | | 14.8  (20)  7.2 – 22.4 | | 2  (18.1)  -6 - 10 | | 22.8 (21.2)  **13.9 – 31.8** | | 14.8 (19.7)  4.2 – 25.3 |
| p-value | 0.72 | | | 0.045 | | | 0.064 | | | | 0.031 | | | | 0.11 | | |
|  | | | | | | | | | | | | | | | | | |
| FACT-G  Mean (SD) | 61.7 (16.1) | | 59.5 (16.5) | 70.5 (16.2) | | 61.7 (19) | 75.7 (15.7) | | 68.9 (18.2) | | 8.8  (14.8)  3.1 – 14.4 | | 2.3  (12.8)  -3.4 – 7.9 | | 13.7 (15.2)  **7.3 – 20.1** | | 9.4 (14.5)  1.8 – 16.8 |
| p-value | NA | | | NA | | | NA | | | | 0.068 | | | | 0.28 | | |
| HCS  Mean (SD) | 29.4 (8.9) | | 30.4 (9.1) | 35.4 (9.1) | | 30.2 (10.7) | 39  (7.6) | | 34.3  (8.5) | | 6.1  (8.1)  2.9 – 9.1 | | -0.24  (9)  -4.2 – 3.7 | | 9.1  (9.3)  5.2 - 13 | | 4.2 (10.0)  -1.1 – 9.5 |
| p-value | NA | | | NA | | | NA | | | | 0.017 | | | | 0.17 | | |
| TOI  Mean (SD) | 53.1 (16.3) | | 51 (18.3) | 65.4 (18.5) | | 53 (22.3) | 73.1 (15.0) | | 61.7 (17.4) | | 11.8 (15.7)  6.3 – 18.3 | | 1.9  (15.1)  -4.7 – 8.6 | | 18.9 (18.0)  **11.3 – 26.6** | | 11.9 (16.7)  3 – 20.8 |
| p-value | NA | | | NA | | | NA | | | | 0.028 | | | | 0.23 | | |
|  | | | | | | | | | | | | | | | | | |
| Physical Well-Being  Mean (SD) | 3.2 (5.6) | | 2.8 (5.1) | 8.2  (5.9) | | 3.8  (6.8) | 10.6  (4.1) | | 7.6  (6.2) | | 4.9  (5.0)  **3.02 – 6.8** | | 1.1  (5.5)  -1.3 – 3.5 | | 6.9  (6.0)  **4.4 – 9.5** | | 4.2  (5.5)  1.4 – 6.9 |
| p-value | NA | | | NA | | | NA | | | 0.026 | | | | | 0.11 | | |
| Functional Well-Being  Mean (SD) | 20.4 (6.2) | 17.7 (6.9) | | 21.8 (6.6) | 18.9 (7.2) | | 23.4  (5.9) | 20.6  (6.9) | | 1.4  (7.6)  -1.5 – 4.2 | | 1.2  (6.1)  -1.5 – 3.8 | | | 2.8  (7.6)  -0.3 - 6 | 2.5  (6.9)  -1 – 6.1 | |
| p-value | NA | | | NA | | | NA | | | | 0.88 | | | | 0.62 | | |
| Emotional Well-Being  Mean (SD) | 9.2 (5.5) | 9.3 (6.5) | | 11.2 (5.6) | | 10  (5.6) | 11.7  (5.7) | | 10.6  (6.9) | | 2  (5.3)  -0.01 - 4 | | | 0.75  (3.7)  -0.8 – 2.3 | 2.7  (5.2)  0.5 – 4.9 | | 2.1  (4.7)  -0.3 – 4.4 |
| p-value | NA | | | NA | | | NA | | | | 0.42 | | | | 0.28 | | |
| Social Well-Being  Mean (SD) | 28.8 (4.1) | | 29.6 (4.6) | 29.3 (3.8) | | 28.9 (5.1) | 29.8  (4.4) | | 30.2  (4.1) | | 0.5  (4.2)  -1.1 – 2.1 | | -0.74 (5.8)  -3.3 – 1.8 | | 1.2  (3)  -0.1 – 2.4 | | 0.6  (4.8)  -1.8 – 2.9 |
| p-value | NA | | | NA | | | NA | | | | 0.36 | | | | 0.59 | | |

*Acronyms: HRQOL – Health-related quality of life; SD – standard deviation; 95%CI – 95% confidence interval; FACT-HEP - Functional Assessment of Cancer Therapy – Hepatobiliary; FACT-G - FACT-HEP - Functional Assessment of Cancer Therapy – General; HCS - hepatobiliary cancer subscale; TOI – Trial Outcome Index; NA – Not Applicable; Resp – responder, NR non-responder*
