# Supplementary material for: Celiac plexus radiosurgery for retroperitoneal pain in advanced cancer: a pre-specified secondary analysis of health-related quality of life in a phase II single-arm trial
Source: eClinicalMedicine. 2026 May 7;95:103968. doi: 10.1016/j.eclinm.2026.103968 (PMC13186082; doi:10.1016/j.eclinm.2026.103968)
Supplement: Supplementary Table 3 [file mmc2.docx]

**Supplementary Table 3. Shapiro-Wilk Normality Tests for Continuous Variables**

| **Variable** | **Analysis Context** | **Normality Test Applied** | **Shapiro-Wilk W** | **p-value** | **Distribution** |
| --- | --- | --- | --- | --- | --- |
| FACT-Hep total score (baseline) | Comparison of baseline scores between responders and non-responders | Shapiro-Wilk | 0.92 | 0.005 | **Non-normal** |
| FACT-G total score (baseline) | Comparison of baseline scores between responders and non-responders | Shapiro-Wilk | 0.92 | 0.003 | **Non-normal** |
| Physical Well-Being (PWB) subscale (baseline) | Comparison of baseline subscale scores between responders and non-responders | Shapiro-Wilk | 0.95 | 0.067 | **Normal** |
| Social/Family Well-Being (SWB) subscale (baseline) | Comparison of baseline subscale scores between responders and non-responders | Shapiro-Wilk | 0.94 | 0.024 | **Non-normal** |
| Emotional Well-Being (EWB) subscale (baseline) | Comparison of baseline subscale scores between responders and non-responders | Shapiro-Wilk | 0.93 | 0.006 | **Non-normal** |
| Functional Well-Being (FWB) subscale (baseline) | Comparison of baseline subscale scores between responders and non-responders | Shapiro-Wilk | 0.97 | 0.26 | **Normal** |
| Hepatobiliary Cancer Subscale (HCS) (baseline) | Comparison of baseline subscale scores between responders and non-responders | Shapiro-Wilk | 0.97 | 0.31 | **Normal** |
| Trial Outcome Index (TOI) (baseline) | Comparison of baseline scores between responders and non-responders | Shapiro-Wilk | 0.94 | 0.02 | **Non-normal** |
| FACT-Hep change score at 3 weeks | One-sample test of change from baseline at 3 weeks | Shapiro-Wilk | 0.98 | 0.58 | **Normal** |
| FACT-Hep change score at 6 weeks | One-sample test of change from baseline at 6 weeks | Shapiro-Wilk | 0.96 | 0.21 | **Normal** |
| BPI average pain score (baseline) | Comparison between responders and non-responders; regression covariate | Shapiro-Wilk* | 0.91 | 0.001 | **Non-normal** |
| BPI average pain score change at 3 weeks | One-sample test of change from baseline at 3 weeks | Shapiro-Wilk* | 0.96 | 0.13 | **Normal** |
| Opioid consumption, mg/day morphine equivalent (baseline) | Comparison between responders and non-responders; regression covariate | Shapiro-Wilk* | 0.77 | <0.0001 | **Non-normal** |
| Overall survival (days) | Kaplan-Meier analysis and group comparison | Shapiro-Wilk | 0.78 | <0.0001 | **Non-normal** |

Abbreviations: BPI = Brief Pain Inventory; EWB = Emotional Well-Being; FACT-G = Functional Assessment of Cancer Therapy – General; FACT-Hep = Functional Assessment of Cancer Therapy – Hepatobiliary; FWB = Functional Well-Being; HCS = Hepatobiliary Cancer Subscale; PWB = Physical Well-Being; SWB = Social/Family Well-Being; TOI = Trial Outcome Index.

* Normality test results for these variables are not shown as they were specified a priori to follow a non-normal distribution based on known clinical characteristics (right-skewed); Wilcoxon rank-sum or signed-rank tests were applied throughout.

† Confirmed non-normal distribution; Wilcoxon rank-sum or signed-rank test applied.
