# Supplementary material for: Celiac plexus radiosurgery for retroperitoneal pain in advanced cancer: a pre-specified secondary analysis of health-related quality of life in a phase II single-arm trial
Source: eClinicalMedicine. 2026 May 7;95:103968. doi: 10.1016/j.eclinm.2026.103968 (PMC13186082; doi:10.1016/j.eclinm.2026.103968)
Supplement: Supplementary Table 4 [file mmc3.docx]

**Supplementary Table 4. Statistical Tests Applied**

| **Variable** | **Analysis Context** | **Test Applied** | **p-value** |
| --- | --- | --- | --- |
| ***Comparison of those included in the HRQOL analysis versus those not included*** | | | |
| Gender | Comparison between 2 groups | Chi-square test | 0.88 |
| Primary Diagnosis | Comparison between 2 groups | Fisher’s Exact Test | 0.41 |
| ECOG | Comparison between 2 groups | Fisher’s Exact Test | 0.59 |
| Prior lines systemic therapy | Comparison between 2 groups | Chi-square test | 0.71 |
| CP invasion | Comparison between 2 groups | Fisher’s Exact Test | 0.61 |
| Survival | Comparison between 2 groups | Wilcoxon Two-Sample Test | 0.0002 |
| Baseline pain score | Comparison between 2 groups | Wilcoxon Two-Sample Test | 0.11 |
| Baseline opioid | Comparison between 2 groups | Wilcoxon Two-Sample Test | 0.19 |
| Baseline FACT-HEP total scores | Comparison between 2 groups | Wilcoxon Two-Sample Test | 0.16 |
| ***Intra-group comparisons*** | | | |
| FACT-Hep change score from baseline at 3- and 6- weeks | Comparison to 3-week score  Comparison to 6-week score | Student T-test  Student T-test | 0.0017  <0.0001 |
| FACT-G change score from baseline at 3- and 6- weeks | Comparison to 3-week score  Comparison to 6-week score | Student T-test  Student T-test | 0.0043  <0.0001 |
| Physical Well-Being (PWB) change score from baseline at 3- and 6- weeks | Comparison to 3-week score  Comparison to 6-week score | Student T-test  Student T-test | 0.0001  <0.0001 |
| Social/Family Well-Being (SWB) change score from baseline at 3- and 6- weeks | Comparison to 3-week score  Comparison to 6-week score | Student T-test  Student T-test | 0.96  0.13 |
| Emotional Well-Being (EWB) change score from baseline at 3- and 6- weeks | Comparison to 3-week score  Comparison to 6-week score | Student T-test  Student T-test | 0.030  0.0028 |
| Functional Well-Being (FWB) change score from baseline at 3- and 6- weeks | Comparison to 3-week score  Comparison to 6-week score | Student T-test  Student T-test | 0.19  0.020 |
| Hepatobiliary Cancer Subscale (HCS) change score from baseline at 3- and 6- weeks | Comparison to 3-week score  Comparison to 6-week score | Student T-test  Student T-test | 0.011  <0.0001 |
| Trial Outcome Index (TOI) change score from baseline at 3- and 6- weeks | Comparison to 3-week score  Comparison to 6-week score | Student T-test  Student T-test | 0.0010  <0.0001 |
| ***Responders versus Non-Responders Baseline Characteristics*** | | | |
| Gender | Comparison between 2 groups | Chi-square test | 0.96 |
| Primary Diagnosis | Comparison between 2 groups | Fisher’s Exact Test | 0.35 |
| ECOG | Comparison between 2 groups | Fisher’s Exact Test | 0.071 |
| Prior lines systemic therapy | Comparison between 2 groups | Chi-square test | 0.61 |
| CP invasion | Comparison between 2 groups | Fisher’s Exact Test | 0.13 |
| Survival | Comparison between 2 groups | Wilcoxon Two-Sample Test | 0.089 |
| Baseline pain score | Comparison between 2 groups | Wilcoxon Two-Sample Test | 0.035 |
| Baseline opioid | Comparison between 2 groups | Wilcoxon Two-Sample Test | 0.23 |
| **Responders** | | | |
| FACT-Hep change score from baseline at 3- and 6- weeks | Comparison to 3-week score  Comparison to 6-week score | Student T-test  Student T-test | 0.0004  <0.0001 |
| FACT-G change score from baseline at 3- and 6- weeks | Comparison to 3-week score  Comparison to 6-week score | Student T-test  Student T-test | 0.0034  0.0002 |
| Physical Well-Being (PWB) change score from baseline at 3- and 6- weeks | Comparison to 3-week score  Comparison to 6-week score | Student T-test  Student T-test | <0.0001  <0.0001 |
| Social/Family Well-Being (SWB) change score from baseline at 3- and 6- weeks | Comparison to 3-week score  Comparison to 6-week score | Student T-test  Student T-test | 0.53  0.073 |
| Emotional Well-Being (EWB) change score from baseline at 3- and 6- weeks | Comparison to 3-week score  Comparison to 6-week score | Student T-test  Student T-test | 0.052  0.016 |
| Functional Well-Being (FWB) change score from baseline at 3- and 6- weeks | Comparison to 3-week score  Comparison to 6-week score | Student T-test  Student T-test | 0.34  0.076 |
| Hepatobiliary Cancer Subscale (HCS) change score from baseline at 3- and 6- weeks | Comparison to 3-week score  Comparison to 6-week score | Student T-test  Student T-test | 0.0004  <0.0001 |
| Trial Outcome Index (TOI) change score from baseline at 3- and 6- weeks | Comparison to 3-week score  Comparison to 6-week score | Student T-test  Student T-test | 0.0002  <0.0001 |
| **Non-Responders** | | | |
| FACT-Hep change score from baseline at 3- and 6- weeks | Comparison to 3-week score  Comparison to 6-week score | Student T-test  Student T-test | 0.61  0.009 |
| FACT-G change score from baseline at 3- and 6- weeks | Comparison to 3-week score  Comparison to 6-week score | Student T-test  Student T-test | 0.42  0.017 |
| Physical Well-Being (PWB) change score from baseline at 3- and 6- weeks | Comparison to 3-week score  Comparison to 6-week score | Student T-test  Student T-test | 0.38  0.0046 |
| Social/Family Well-Being (SWB) change score from baseline at 3- and 6- weeks | Comparison to 3-week score  Comparison to 6-week score | Student T-test  Student T-test | 0.56  0.61 |
| Emotional Well-Being (EWB) change score from baseline at 3- and 6- weeks | Comparison to 3-week score  Comparison to 6-week score | Student T-test  Student T-test | 0.35  0.089 |
| Functional Well-Being (FWB) change score from baseline at 3- and 6- weeks | Comparison to 3-week score  Comparison to 6-week score | Student T-test  Student T-test | 0.37  0.15 |
| Hepatobiliary Cancer Subscale (HCS) change score from baseline at 3- and 6- weeks | Comparison to 3-week score  Comparison to 6-week score | Student T-test  Student T-test | 0.90  0.11 |
| Trial Outcome Index (TOI) change score from baseline at 3- and 6- weeks | Comparison to 3-week score  Comparison to 6-week score | Student T-test  Student T-test | 0.54  0.012 |
| ***Responders versus Non-Responders*** | | | |
| FACT-Hep change score from baseline at 3- and 6- weeks | Baseline scores  3-week scores  6-week scores  3-week change scores  6-week change scores | Wilcoxon Two-Sample Test | 0.72  0.045  0.068  0.031  0.11 |
| FACT-G change score from baseline at 3- and 6- weeks | 3-week change scores  6-week change scores | Wilcoxon Two-Sample Test | 0.068  0.28 |
| Physical Well-Being (PWB) change score from baseline at 3- and 6- weeks | 3-week change scores  6-week change scores | Wilcoxon Two-Sample Test | 0.026  0.11 |
| Social/Family Well-Being (SWB) change score from baseline at 3- and 6- weeks | 3-week change scores  6-week change scores | Wilcoxon Two-Sample Test | 0.36  0.59 |
| Emotional Well-Being (EWB) change score from baseline at 3- and 6- weeks | 3-week change scores  6-week change scores | Wilcoxon Two-Sample Test | 0.42  0.28 |
| Functional Well-Being (FWB) change score from baseline at 3- and 6- weeks | 3-week change scores  6-week change scores | Wilcoxon Two-Sample Test | 0.88  0.62 |
| Hepatobiliary Cancer Subscale (HCS) change score from baseline at 3- and 6- weeks | 3-week change scores  6-week change scores | Wilcoxon Two-Sample Test | 0.017  0.17 |
| Trial Outcome Index (TOI) change score from baseline at 3- and 6- weeks | 3-week change scores  6-week change scores | Wilcoxon Two-Sample Test | 0.028  0.23 |

Abbreviations:ECOG – Eastern Cooperative Group performance status; CP – celiac plexus; BPI = Brief Pain Inventory; EWB = Emotional Well-Being; FACT-G = Functional Assessment of Cancer Therapy – General; FACT-Hep = Functional Assessment of Cancer Therapy – Hepatobiliary; FWB = Functional Well-Being; HCS = Hepatobiliary Cancer Subscale; PWB = Physical Well-Being; SWB = Social/Family Well-Being; TOI = Trial Outcome Index.
