# Supplementary material for: Celiac plexus radiosurgery for retroperitoneal pain in advanced cancer: a pre-specified secondary analysis of health-related quality of life in a phase II single-arm trial
Source: eClinicalMedicine. 2026 May 7;95:103968. doi: 10.1016/j.eclinm.2026.103968 (PMC13186082; doi:10.1016/j.eclinm.2026.103968)
Supplement: CPSRS.Protocol [file mmc4.pdf]

BMJ Open

Coeliac plexus radiosurgery for pain management in patients with advanced cancer : study protocol for a phase II clinical trial

Galia Jacobson ,[1,2] Ronen Fluss,[3] Amira Dany-BenShushan,[4] Talia Golan,[5,6] Tikva Meron,[5] Camilla Zimmermann ,[7] Laura A Dawson,[8] Aisling Barry,[8] Marcin Miszczyk ,[9] Michael Buckstein,[10] Dayssy Diaz Pardo,[11] Artur Aguiar,[12] Liat Hammer,[1,13] Adam P Dicker,[14] Maoz Ben-Ailan,[1] Ofir Morag,[15] David Hausner,[16] Zvi Symon,[1,6] Yaacov R. Lawrence [1,6,14]

To cite: Jacobson G, Fluss R, Dany-BenShushan A, *et al*. Coeliac plexus radiosurgery for pain management in patients with advanced cancer : study protocol for a phase II clinical trial. *BMJ Open* 2022;**12**:e050169. doi:10.1136/bmjopen-2021-050169

► Prepublication history and additional supplemental material for this paper are available online. To view these files, please visit the journal online (http://dx.doi.org/10.1136/bmjopen-2021-050169).

Received 14 February 2021
Accepted 08 February 2022

Check for updates

© Author(s) (or their employer(s)) 2022. Re-use permitted under CC BY-NC. No commercial re-use. See rights and permissions. Published by BMJ.

For numbered affiliations see end of article.

Correspondence to
Dr Yaacov R. Lawrence; yaacov.lawrence@sheba.health.gov.il

ABSTRACT

Introduction Pancreatic cancer is characterised by severe mid-back and epigastric pain caused by tumour invasion of the coeliac nerve plexus. This pain is often poorly managed with standard treatments. This clinical trial investigates a novel approach in which high-dose radiation (radiosurgery) is targeted to the retroperitoneal coeliac plexus nerve bundle. Preliminary results from a single institution pilot trial are promising: pain relief is substantial and side effects minimal. The goals of this study are to validate these findings in an international multisetting, and investigate the impact on quality of life and functional status among patients with terminal cancer.

Methods and analysis A single-arm prospective phase II clinical trial. Eligible patients are required to have severe coeliac pain of at least five on the 11-point BPI average pain scale and Eastern Cooperative Oncology Group performance status of two or better. Non-pancreatic cancers invading the coeliac plexus are also eligible. The intervention involves irradiating the coeliac plexus using a single fraction of 25 Gy. The primary endpoint is the complete or partial pain response at 3 weeks. Secondary endpoints include pain at 6 weeks, analgesic use, hope, qualitative of life, caregiver burden and functional outcomes, all measured using validated instruments. The protocol is expected to open at a number of cancer centres across the globe, and a quality assurance programme is included. The protocol requires that 90 evaluable patients" be accrued, based upon the assumption that a third of patients are non-evaluable (e.g. due to death prior to 3-weeks post-treatment assessment, or spontaneous improvement of pain pre-treatment), it is estimated that a total of 120 patients will need to be accrued. Supported by Gateway for Cancer Research and the Israel Cancer Association.

Ethics and dissemination Ethic approval for this study has been obtained at eight academic medical centres located across the Middle East, North America and Europe. Results will be disseminated through conference presentations and peer-reviewed publications.

Trial registration number NCT03323489.

Strengths and limitations of this study

- ► The trial focuses on an urgent unmet clinical need: patients with advanced cancer whose pain is refractory to narcotic analgesics.
- ► The technique being tested is non-invasive, and can be easily implemented using contemporary, widely available radiotherapy equipment. Results from the pilot study are promising.
- ► The trial will provide a broad insight into the functional and social aspects of pain's impact on patients' lives through the use of a range of validated instruments assessing quality of life, functional outcomes, hope and caregiver burden. Moreover, the trial will investigate whether the intervention is able to reverse these changes.
- ► The primary limitation of the study is the use of pain as both an eligibility criterion and the primary endpoint. Pain is a complex subjective experience that is difficult to measure, and somewhat unstable. The concurrent use of opioid analgesics will complicate the efficacy assessment.
- ► The non-randomised design is a limitation.

INTRODUCTION

Coeliac pain in pancreatic and other malignancies

Pain is a characteristic feature of pancreatic cancer, both at diagnosis[1 2] and in terminal disease.[3] Pain is more frequently seen in tumours of the body and tail, than the head of pancreas.[4] Almost one-third of patients define the pain as being of at least moderate to severe intensity at diagnosis,[1] and one-third of subjects report poor pain relief despite oral analgesics.[1] The pain is associated with a poor quality of life and depression.[1 5 6] Pancreatic cancer is common, with over 50 000 cases annually in each of the USA[7] and Europe,[8] moreover, incidence appears to be rising.[9]

Pancreatic cancer pain typically emanates from the mid-back and radiates to the epigastric area, termed the midline retroperitoneal pain syndrome.[10] Tumour invasion of the coeliac nerve plexus is thought to be the cause the pain.[11] Other tumour types metastatic to the retroperitoneum/coeliac axis region may induce a similar pain syndrome.

Current palliative approaches for the retroperitoneal pain syndrome include the use of analgesics, coeliac nerve block and systemic chemotherapy. Opioid analgesics (ie, morphine, oxycodone, fentanyl) are commonly used in pancreatic cancer,[12] yet the high doses frequently required are associated with side effects including constipation, sedation, pruritus and nausea.[11] These side effects may prevent patients from obtaining adequate pain relief.

For refractory coeliac pain, invasive procedures may be considered, especially 'coeliac plexus neurolysis' and 'coeliac plexus block', performed either via a transcutaneous or transoesophageal approach. The chemical ablation or numbing of nerve fibres transmitting signals from the intra-abdominal viscera to higher nerve levels, aims to alleviate pain. Some trials have shown significant pain reduction and lower opioid consumption following the procedure,[13–17] but other data did not suggest an improved quality of life,[18] furthermore, the degree of pain relief appears to be modest. A recent randomised trial of endoscopic coeliac plexus neurolysis failed to demonstrate a reduction in pain compared with analgesics alone.[17 19]

Systemic chemotherapy is another option. Both gemcitabine[20] and combination treatment with oxaliplatin, irinotecan, fluorouracil and leucovorin[21] have been shown to reduce pain and improve quality of life in pancreatic cancer, however, these treatments are associated with side effects, and the analgesic benefit is often short-lived.

Hence pain remains a substantial problem for many patients with pancreatic cancer and other malignancies of the upper abdomen involving the coeliac plexus. The pancreatic cancer pain syndrome has been identified by Prof Nathan Cherny for the European Society for Medical Oncology as a uniquely 'difficult pain problem'.[10] Progress has been limited, as reflected by a population-based study from Australia published in 2016, that identified 'pain' as a frequently unmet need among people with pancreatic cancer.[22]

GROSS AND NEUROANATOMY OF THE COELIAC PLEXUS

The coeliac plexus is a dense network of interconnecting nerve fibres connecting the coeliac, superior mesenteric and renal ganglia. Anatomically it extends over the anterolateral surface of the aorta, around the origins of the coeliac and superior mesenteric arteries.[16] The coeliac plexus demonstrates considerable variability in size and position. Nonetheless 94% of the coeliac ganglia are located at the level of T12 or L1 vertebrae.

The coeliac plexus is composed of both efferent and afferent, sympathetic and parasympathetic nerve fibres. Of key importance to this protocol, the visceral afferent fibres that carry nociceptive stimuli from the upper abdominal viscera (including the pancreas, liver, biliary tract, gallbladder and the small bowel) pass through the coeliac plexus before terminating in the dorsal horn of the thoracic cord. Hence the coeliac plexus represents the main target point of pain transmission from the upper abdominal organs.

CONTEMPORARY USE OF RADIATION FOR PALLIATION IN PANCREATIC CANCER

The contemporary use of radiation as a palliative modality in pancreatic cancer is limited. Several small retrospective studies[23–26] using various radiation doses, have suggested that radiotherapy is a safe and effective palliative modality in pancreatic cancer; however, the studies size and retrospective nature, limit the generalisable of their findings. One retrospective analysis recommended a dose of 30 Gy in 10 fractions.[25]

PILOT STUDY

A small pilot study performed at the Sheba Medical Center between 2013 and 2017 examined the palliative role of coeliac plexus radiosurgery (ClinicalTrials.gov NCT02356406). The radiation dose was originally 45 Gy in five fractions, but later amended to a single fraction of 25 Gy.[27] The primary endpoint was pain relief 3 weeks' post-treatment. Twenty-five subjects underwent treatment with a single fraction, of whom 18 were evaluable. Median age of 68 years, median Eastern Cooperative Oncology Group (ECOG) 1, 89% had pancreatic cancer. The pain relief following intervention was substantial, with minimal side effects. The manuscript with full results was submitted for publication.

KEY HYPOTHESES

- Ablative radiation targeted to the coeliac plexus will alleviate pain.
- Decreased pain will be associated with improved patient functionality, quality of life and hope.
- Decreased pain will be associated with decreased caregiver burden.

Patients with pancreatic cancer suffer from impaired functioning and quality of life. We hypothesise coeliac plexus tumour infiltration to be the fundamental cause of pain, and consequent decreased functionality, decreased quality of life, increased opioid usage, impaired hope and resultant caregiver burden. By intervening at an early stage in the pathway, that is, blocking of coeliac plexus induced pain, we hypothesise that we will be able to reverse these negative processes, reducing suffering and consequently improve patients' hope and potentially their ability to undergo further treatments. Figure 1 shows a model of how radiosurgical intervention impacts patient well-being. Conversely, we acknowledge that some

Jacobson G, *et al*. *BMJ Open* 2022;**12**:e050169. doi:10.1136/bmjopen-2021-050169

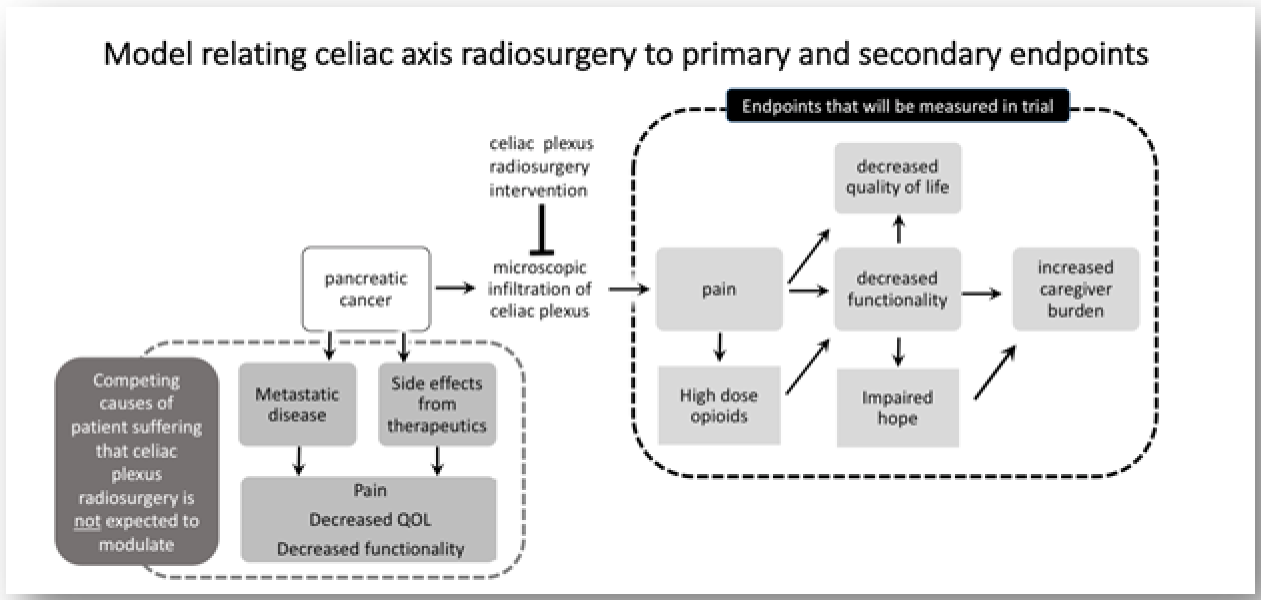

Figure 1 Model of how radiosurgical intervention is hypothesised to impacts patient well-being. QOL, quality of life.

pain and suffering is unrelated to the coeliac plexus, for example, pain resultant from liver metastases and peripheral neuropathy resultant from cytotoxic chemotherapy. These symptoms are not expected to be improved by our intervention (coeliac plexus radiotherapy) and hence are identified in our model as competing causes of suffering.

METHODS AND ANALYSIS

This protocol described a multicentre, single-arm phase II interventional trial, assessing a new radiation technique for pain management. Patients will be recruited in the oncology departments of participating hospitals.

ELIGIBILITY CRITERIA

Key inclusion criteria

- Age ≥18 years.
- A malignancy that is metastatic or unresectable.
- Severe retroperitoneal pain syndrome (radiates from the lower back to the upper abdomen, belt-like distribution), intensity of at least 5 on 11 point Brief Pain Inventory (BPI, average pain) scale despite analgesic use.
- Anatomical involvement of the coeliac plexus, as defined by either:
  - Any Pancreatic cancer.
  - Any other cancer that on imaging demonstrates either: gross involvement of the coeliac blood vessels or coeliac plexus on imaging OR haziness around the coeliac blood vessels, that typically implies tumour engulfment.
- Prior chemotherapy or biological treatment is allowed, but any active oncological treatment should be stopped at least 6 days prior to radiation therapy and renewed at least 6 days following radiation therapy.

Key exclusion criteria

- Patients who are well balanced in terms of pain control.
- Patients with life expectancy <8 weeks.
- Significant comorbidities.
- Patients with ECOG Performance status 3 or 4.
- Previous radiotherapy to upper abdomen.
- Conditions associated with increased side effects to radiotherapy (eg, inflammatory bowel disease, scleroderma).

Of note, previous use of a coeliac plexus block/neurolysis (or similar procedure) is allowed and does not interfere with the trial, but will be recorded.

INTERVENTION

Figure 2 shows a schema of the study recruitment process and overall study design. Patients should be simulated supine with arms above the head on a chest board, with oral and intravenous contrast administered. The three-dimensional simulation CT scan should span from the carina until at least L5-S1 with a slice thickness, 3 mm or less. A motion management technique (eg, 4 Dimensional - Planning Organ at Risk Volume, 4D-PRV) approach, breath-hold or gating) is required.

CONTOURING

The coeliac plexus is not visible on conventional imaging. The anterior and medial aspects of the aorta from the levels of the T12–L2 vertebrae inclusive are contoured as a surrogate structure (figure 3). The inclusion of tumour immediately adjacent to the coeliac plexus, and the prescribed dose to such tumour, is left to the physician's discretion but will be recorded. The following normal structures need to be contoured: spinal cord, liver,

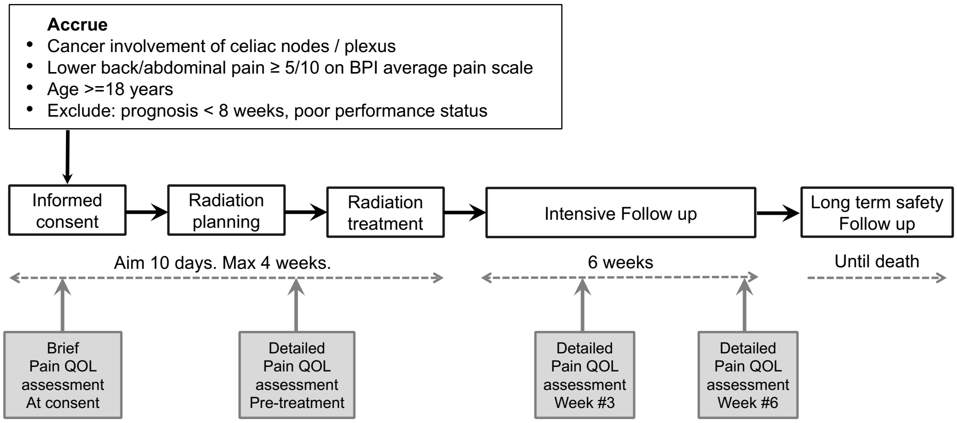

Figure 2 Trial schema. QOL, quality of life.
Q0L: Quality Of Life

kidneys, stomach-duodenum and small bowel in accordance with Radiation Therapy Oncology Group, RTOG guidelines.[23] The duodenum is the critical structure of especial concern due to its proximity to the coeliac plexus. The stomach, small bowel, large bowel and sometimes the oesophagus must also be considered.

DOSE PRESCRIPTION AND CONSTRAINTS

The prescription dose to the coeliac plexus is 25 Gy. The duodenum lies in close proximity to the coeliac plexus, yet is very sensitive to radiation. To overcome this challenge a dose-painting technique was developed; briefly, bowel loops are to be precisely contoured. Within the coeliac plexus contour, voxels within 0.5 cm of bowel will be prescribed 10 Gy (modPTV 10), those at least 0.5 cm, but no more than 1 cm from the bowel, will be prescribed 15 Gy (modPTV 15). Voxels at least 1 cm from the bowel within the coeliac plexus itself will be prescribed 25 Gy (modPTV 25), and those within the 0.5 cm isotropic expansion of the coeliac plexus 20 Gy (modPTV 20).

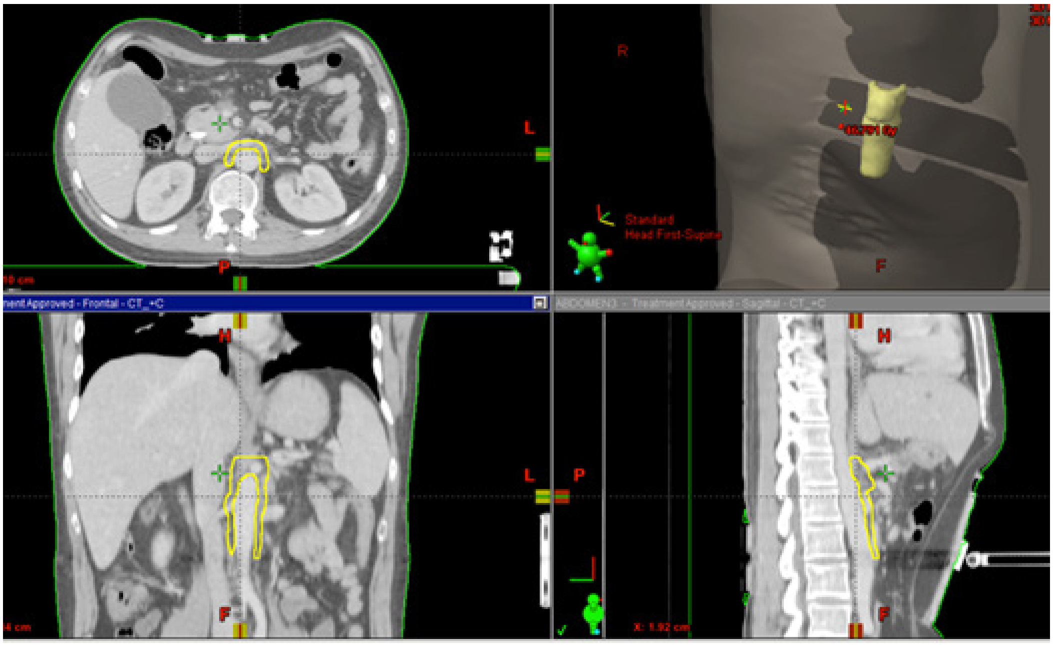

Figure 3 Coeliac plexus target deliniation anterior and medial aspects of the aorta contoured from top of T12 to bottom of L2, a surrogate structure for the coeliac plexus (yellow structure).

 Jacobson G, *et al. BMJ Open* 2022;**12**:e050169. doi:10.1136/bmjopen-2021-050169

Table 1 Acceptable and unacceptable variations in D2% and D95% of each PTV

Name of structure	Typical mean dose	D2% aim	D2% acceptable deviation	D2% unacceptable deviation	D95% aim	95% acceptable deviation	D95% unacceptable deviation
modPTV25	25.5±2	26	≤2 Gy more/less than 'D2% aim'	≥2 Gy more/less than 'D2% aim'	24	≤2 Gy more/ less than 'D95% aim'	≥2 Gy more/less than 'D95% aim'
modPTV20	22±2	24.5			19		
modPTV15	17±1.5	20			14		
modPTV10	12±1	14			10		

PTV, Planning Target Volume.

Acceptable and unacceptable variations in D2% and D95% of each PTV are detailed in table 1.

Dose constraint's for normal organs are provided in table 2. In general, the 'organs at risk' dose limits have a higher priority than the target structure modPTVs. When calculating maximum dose, very small volumes <0.3 cc (ie, the hot but very thin tail of the Dose-Volume Histogram, DVH) may be ignored.

TREATMENT DELIVERY

Treatment will be delivered with a megavoltage LINAC, preferably within ten days of simulation. It is essential that image-guided radiation therapy techniques be employed. As a minimum, a cone-beam CT should be performed in the treatment position prior to treatment. It is recommended to give oral contrast or water 20 min prior to treatment in order to visualise the duodenum better. The conebeam CT should be matched on the small bowel/aorta.

PROPHYLACTIC ANTIEMETIC TREATMENT

All patients are recommended to received prophylactic antiemetic medication, such as a single dose of combined netupitant/palonosetron, 8 mg dexamethasone and a proton pump inhibitor (mandatory, continue for 4 weeks). As an alternative netupitant/palonosetron may be replaced with ondansetron 8 mg two times per day for 2 days.

CONCOMITANT MEDICATIONS

Anticancer treatments including chemotherapy, targeted anticancer agents, and immunotherapy should be not be administered at least 6 days prior to and 6 days following treatment. All other medications may be continued during the treatment.

PAIN MEDICATIONS

No limitations are placed on the use of pain medications before or after treatment. The majority of subjects on this protocol will be receiving substantial doses of opioid medications, both long acting and short acting. The use, type and dosage of opioids will be carefully recorded and converted into intravenous morphine milligram equivalents.

A palliative nurse is the responsible for maintaining weekly contact with patients, assessing pain levels and modifying opioid use as appropriate. These contacts should preferably commence prior to receiving radiation therapy. Patient should be educated to take breakthrough medication only as needed for pain, not on a regular basis, and to advise the team if pain levels decrease so that long-term opioid levels can be modified.

QUALITY ASSURANCE PROCEDURES

This trial incorporates several levels of quality assurance: (1) a benchmark case, requiring contouring and treatment planning; (2) an online exam to ascertain the subinvestigator's understanding of the protocol; (3) the initial three cases require pre-treatment authorisation by the principal investigator, and other cases at the investigators discretion and (4) post-treatment quality assurance at the conclusion of the trial. Furthermore, within each institution, peer-to-peer review is recommended.

Table 2 Dose constraints for normal organs

	Recommended	Acceptable deviation
Each kidney	Mean dose ≤5.5 Gy.	One individual kidney has a mean dose of <7.5 Gy, but both functional kidneys together have a mean dose of ≤5.5 Gy.
Bowel	Less than 1 cc receive 11 Gy.	No more than 5 cc receive over 12 Gy. Max 15 Gy.
Liver	700 cc receive less than 10 Gy.	
Spinal cord	Max. dose 10 Gy	Less than 1 cc receive 11 cc.

Table 3 Validated instruments to assess patients' pain level, quality of life, functional status, hope level and caregiver burden

	Instrument	Comments
Pain intensity	Brief Pain Inventory Short form (BPI-SF)	Developed by Cleeland for measuring pain related to cancer, the BPI-SF incorporates an 11 point (0–10) numerical rating scale for pain. A two-point decrease on the 0 to 11 pain intensity numerical rating scale is considered equivalent to a 30% change in pain intensity,[36] and represents 'notable improvement'. The BPI-SF also includes measures of pain interference with daily function.[34]
Quality of life	FACT-Hep	A 45-item self-report instrument was developed specifically to measure HRQoL in patients with hepatobiliary cancer.[3 4 6] It consists of the backbone FACT-G questionnaire, which assesses symptoms and other HRQoL concerns across four dimensions (physical well-being (seven items), social/family well-being (seven items), emotional well-being (six items)and functional well-being (seven items)) together with an 18-item disease-specific hepatobiliary cancer subscale (HCS). The HCS assesses back and stomach pain, gastrointestinal symptoms, anorexia, weight loss and jaundice in patients with hepatobiliary cancers.
Side effects/toxicity	Common Terminology Criteria for Adverse Events V.4.03	A standardised system to quantify or grade the severity of adverse events that occur with drug treatment or from medical devices, developed by the CTEP of the NCI[37]
Hope	The Goal Assessment Scale	The 'Goal Assessment Scale' contains six items. Three items measure pathways thinking, and three items measure agency thinking. Participants respond to each item using an 8-point scale ranging from definitely false to definitely true and the scale takes only a few minutes to complete.[10]
Functional assessment	6 min walk test.<br>Handgrip strength test.	These test how far the patient can walk in 6 min, and their maximal hand-grip strength. Both tests have been validated in patients with cancer.[11 12] Standardised methods will be used.
Caregiver burden	Short version of the Zarit Burden Interview.	Caregiver burden is commonly used to describe the multiple dimensions of distress that result from an imbalance between care demands and the availability of resources to meet those demands.[13] We will use a validated shortened 12 item version of the original interview.[14 15]
Activity	Measured by wearable fitness tracker.	This will be measured by an electronic wearable device, for example, manufactured by Garmin (Olathe, Kansas) or Fitbit (San Francisco, California), from the time of registration until the 6 weeks follow-up visit. Of note, this is an optional experimental endpoint that both institutions and individuals can decide to opt out of.

The caregiver burden questionnaire is completed by an accompanying caregiver.
CTEP, Cancer Therapy Evaluation Program; FACT-G, Functional Assessment of Cancer Therapy - General; HRQoL, Health Related Quality of Life; NCI, National Cancer Institute.

PATIENT REPORTED OUTCOMES/ HEALTH-RELATED QUALITY OF LIFE INSTRUMENTS TO BE USED

A range of validated instruments will be used to assess patients' pain level, quality of life, functional status, hope level and caregiver burden (table 3). An additional experimental instrument that will be offered to participants is use of a fitness tracker (also called an activity tracker). The device will record daily step count and sleep hours on a daily basis.

BIOSTATISTICAL CONSIDERATIONS

Definition of evaluable patient

An evaluable patient is defined as a patient, eligible for enrolment per the defined criteria, who has received the therapy per protocol and remains alive until the 3-week post-treatment pain and quality of life assessment. A further evaluability criterion is that BPI average pain remains greater than or equal to 4 on the 11-point scale at the assessment immediately before the first treatment (the eligibility level cut-off at recruitment is 5). This is required to ensure that all patients have pre-treatment pain at a sufficient level to allow detection of pain relief following treatment. An additional criterion is that any reduction between the screening BPI and the BPI immediately before the treatment is no more than two. Toxicity will be assessed in all patients, even those who do not complete the 3-week post-treatment assessment.

SAMPLE SIZE

The authors consider the radiosurgical procedure to be justified if at least 40% will have a successful outcome. Assuming that the true response rate is 60%, a trial with 100 patients will have a 97% chance of demonstrating at a one-sided statistical significance level of 2.5% that the response rate is at least 40%. This calculation assumes and takes into account that 10% of patients will be non-evaluable. Therefore, during the trial, the number of evaluable patients will be monitored, and a minimum of 90 evaluable patients will be entered. A principal aim of the study is to estimate the pain response rate. With a 60% success rate and 100 patients entered, the SE of the estimated response rate will be ~5%, and the 95% CI will be approximately ±10% around the point estimate. It was noted mid-trial that approximately a third of patients were non-evaluable, hence a larger number (approx. 120) would be needed to achieve 90 evaluable patients.

ENDPOINTS

The primary endpoint is complete or partial pain response, based on the BPI average pain 11-point scale, defined as a decrease between the score immediately before treatment and 3 weeks' post-treatment, that is, two or more, and is also at least two more than any decrease between registration and the score immediately before treatment. Some patients find it difficult to verbally express their pain from 'zero to ten' (Numeric Rating Scale, NRS), for such patients it may be useful to use the following Visual Analogue Scale (VAS). Most studies have found the NRS to correlate well with the VAS,[28–30] however, it is best to be consistent in their use for each individual patient.[31]

Secondary endpoints include changes from baseline to both 3-week and 6-week post-treatment in the following metrics: 'BPI average pain', 'BPI worse pain', 'daily opioid usage' (in mg intravenous morphine equivalent), overall quality of life (FACT-Hep), Hepatobiliary Cancer QOL subscale (a measure of gastrointestinal toxicity), functionality (handgrip, walking, daily step count), use of short-acting opioids for breakthrough pain measured both in morphine-equivalent dose per day and times taken per day.

Exploratory endpoints include a change in caregiver burden (Zarit Burden Interview, short 12-item version), change in Goal Assessment Scale, and change in the number of times short-acting opioids were used for breakthrough pain ('rescue analgesic doses'), averaged over the previous 3 days, sleep as assessed with an activity tracker. Interactions between pain dynamics, the intervention and analgesic use will be assessed both graphically and analytically—using for instance the integrated method used by Mercadant[17]

STATISTICAL ANALYSIS

The response rate will be estimated as the proportion of evaluable patients who achieve a complete or partial pain response. The 95% CIs will be calculated based on the binomial distribution. A statistical test of the null hypothesis that the response rate is 40% (the rate that would be considered large enough to justify the adoption of the treatment assuming minimal toxicity) will be conducted at the one-sided 2.5% level, based on the binomial distribution.

Patients who are still alive but do not provide a 3-week pain assessment will be evaluable and will be included as failures. However, a sensitivity analysis will be added in which patients with no 3-week pain assessment will be excluded. This alternative estimate of the response rate and its CI, and the associated test of the null hypothesis that the response rate is 40%, will be presented.

Two approaches will be taken to analyse the relationship between changes in BPI average pain score and changes in other endpoints: First, patients will be divided into two subgroups: those with a defined pain response and those with no response. Then for each of the other endpoints the mean change in the endpoint at 3 weeks will be computed for the two subgroups and compared using a t-test. Second, the 3-week change in each endpoint will be regressed on the change in the pain score at 3 weeks and the linear slope and correlation coefficients will be estimated. The test of the null hypothesis that there is zero correlation will be tested using the t-test for a linear association.

Exploratory analyses will be performed to identify predictors of response (ie, understand who benefits most from the intervention), and to test for heterogeneity of response rate across centres. Furthermore, mediation effects will be examined, for example, whether functionality is a mediator between pain and caregiver burden.

ETHICS AND DISSEMINATION

The study will be opened at a number of academic radiation oncology departments worldwide. At the time of writing, the study has been approved and opened at: Princess Margaret Cancer Centre, Toronto, Canada; Mount Sinai Hospital, New York, USA; Ohio State University Hospital, Ohio, USA; Instituto Portugues de Oncologia, Porto, Portugal; Assuta Medical Center, Tel Aviv, Israel; Sourasky Medical Center, Tel Aviv, Israel; Maria Sklodowska-Curie National Research Institute of Oncology, Gliwice, Poland. Results will be disseminated through conference presentations and peer-reviewed publications.

INFORMED CONSENT

The patient will be approached and informed about the trial by the investigator and provided with a copy of the patient information and consent form. Patients will be given an adequate amount of time to consider their participation in the trial and will be given an opportunity to ask questions if needed. If the patient decides to participate in the study, they will be asked to provide written consent. All participants are free to withdraw from the study at any time, without any prejudice to future medical treatment. See online supplemental file 1 for the informed consent form.

SAFETY

Adverse events will be recorded using NCI Common Terminology Criteria for Adverse Events V.4.0 . Severe adverse events will be reported urgently to both the IRB and the principal investigator. At three prescribed periods (after 10, 35 and 70 patients accrued), a data and safety monitoring board (DSMB) will review the efficacy and toxicity data. Long term follow-up for up to 2 years will be performed to assess for efficacy and late toxicities.

PROTOCOL AMENDMENTS

Due to the COVID-19 pandemic that erupted in early 2020 the protocol was amended to allow follow-up visits to be performed virtually (eg, over the telephone). The

protocol initially adopted the Adult Hope Scale (AHS). In September 2020, an amendment was made to the protocol to use the 'Goal Assessment Scale' in place of the AHS. This change was made since patients had great difficulty completing the non-specific questions contained within the AHS, some of which were felt to be inappropriate for terminal patients, such as '*I worry about my health'* and '*I've been pretty successful in life'*. The 'Goal Assessment Scale' has been found to predict goal attainment better than the AHS, which measures hope regarding goals in general.[32] The current protocol is dated September 2020 V.1.5.

PATIENT AND PUBLIC INVOLVEMENT

During the single-institution pilot trial, patients expressed satisfaction with both the treatment and trial design, which have been closely replicated in the current multicentre trial. At patients' request we encourage investigators to schedule follow-up visits on days when subjects are already attending the hospital. Several patients expressed interest in an electronic means of gathering pain scores (eg, via smartphone)—and this has been implicated. A detailed patient feedback form is incorporated into the trial at 3-week and6-week post-treatment. Based on our experience that patients are not always willing/able to complete all questionnaires, a priority list has been incorporated into the protocol clarifying that pain and analgesic assessments have priority over functional and hope assessments. Following consent, patients are incorporated in media briefings aiming to boost accrual. Trial subjects have limited life expectancies, hence direct dissemination to participants is inappropriate.

DISCUSSION

The purpose of this protocol is to establish a new treatment for refractory retroperitoneal cancer pain, characteristic of pancreatic neoplasms. Following on from a promising pilot trial, the protocol will examine the treatment in a multicentre international meeting, establishing both toxicity and efficacy data. Through use of extensive secondary measures, we seek to understand the impact of pain on these patients' physical and psycho-social functioning, their caregivers and moreover what happens after the pain improves.

The protocol has a number of limitations. First, the primary endpoint is 'pain level' as measured on the 11-point BPI scale, likewise a 'pain level' of at least five out of ten is an eligibility criteria. Pain is a subjective experience which cannot be objectively measured,[30 33] being influenced by many factors including stress, emotional state[1] and use of analgesics. The protocol uses the widely accepted BPI instrument as a measure of pain,[34] asking patients to focus on the pain location described at baseline. An unexpected concern of the DSMB on reviewing the ongoing trial's data was the instability of pain. Patients have pain recorded at least twice and sometimes three times prior to treatment: at initial meeting with physician, at signing of consent (often on a different day) and within a week prior to treatment (often the day of treatment); the DSMB noted that some patients had spontaneous improvement of pain. The protocol was subsequently amended to categorise such patients as 'unevaluable'.

An additional obstacle is the challenging patient population: based on our pilot trial, we expect that many of the enrolled subjects will have progressed on first-line systemic treatment and hence have a limited life expectancy; in that trial median overall survival at accrual was 3 months. This poses a number of challenges—regarding obtaining long-term follow-up data and the development of multiple new palliative challenges that characterise terminal cancer, including ascites, additional metastases and depression. Hence even if the intervention is efficacious and the retroperitoneal pain improves, this may not be reflected in improved quality of life, functional status or mood.

Ideally, this would have been a randomised phase II trial, possibly with a cross-over design, comparing coeliac plexus radiosurgery with a standard of care—coeliac nerve block or neurolysis. The investigators considered the logistic challenges and expense of running such a trial insurmountable; trials comparing different treatment modalities are complex and frequently accrue poorly.[35]

Author affiliations
[1]Radiation Oncology, Sheba Medical Center, Tel Hashomer, Tel Aviv, Israel
[2]Radiation Oncology, MD Anderson Cancer Center, Houston, Texas, USA
[3]Gertner Institute, Sheba Mediacal Center, Tel Hashomer, Tel Aviv, Israel
[4]Israeli Center for Cardiovascular Research, Sheba Medical Center, Tel Hashomer, Tel Aviv, Israel
[5]Oncology, Sheba Medical Center, Tel Hashomer, Tel Aviv, Israel
[6]Sackler School of Medicine, Tel aviv University, Tel Aviv, Israel
[7]Department of Supportive Care, Princess Margaret Cancer Centre, University Health Network and Department of Medicine, University of Toronto, Toronto, Ontario, Canada
[8]Radiation Oncology, Princess Margaret Hospital Cancer Centre, University of Toronto, Toronto, Ontario, Canada
[9]IIIrd Radiotherapy and Chemotherapy Department, Maria Sklodowska-Curie National Research Institute of Oncology, Gliwice, Poland
[10]Department of Radiation Oncology, Icahn School of Medicine at Mount Sinai, New York, New York, USA
[11]Department of Radiation Oncology, The Ohio State University Medical Center, Columbus, Ohio, USA
[12]Radiation Oncology, Portuguese Institute of Oncology of Porto, Porto, Portugal
[13]Radiatin Oncology, University of Michigan, Ann Arbor, Michigan, USA
[14]Radiation Oncology, Thomas Jefferson University, Philadelphia, Pennsylvania, USA
[15]Cancer Pain Unit, Institute of Oncology, Sheba Medical Center, Tel Aviv, Israel
[16]Cancer Center, Sheba Medical Center, Tel Hashomer, Tel Aviv, Israel

Twitter Yaacov R. Lawrence @LawrenceYaacov

Contributors Trial statistician: RF. Overall PI: YL (employee of Sheba Medical Center, Israel). Clinical coinvestigators including subsite PIs: GJ, ZS, AB, MM, MB, DDP, AA, LH and LD. Trial design (radiation therapy): ZS, MB-A and APD. Trial design (palliative care and pain control): DH, OM, TM and CZ. Trial design (pancreatic cancer): TG Trial design (logistics): AD-B.

Funding This work is supported by a major grant from 'Gateway for Cancer Research' (G-17-100), with additional support from the 'Israel Cancer Association' through a donation from Irma and Simon Gross.

Disclaimer The funding agencies had/have no role in the trial design, data analysis or future publication decisions.

Competing interests None declared.

Patient consent for publication Consent obtained directly from patient(s)

Provenance and peer review Not commissioned; externally peer reviewed.

Supplemental material This content has been supplied by the author(s). It has not been vetted by BMJ Publishing Group Limited (BMJ) and may not have been peer-reviewed. Any opinions or recommendations discussed are solely those of the author(s) and are not endorsed by BMJ. BMJ disclaims all liability and responsibility arising from any reliance placed on the content. Where the content includes any translated material, BMJ does not warrant the accuracy and reliability of the translations (including but not limited to local regulations, clinical guidelines, terminology, drug names and drug dosages), and is not responsible for any error and/or omissions arising from translation and adaptation or otherwise.

Open access This is an open access article distributed in accordance with the Creative Commons Attribution Non Commercial (CC BY-NC 4.0) license, which permits others to distribute, remix, adapt, build upon this work non-commercially, and license their derivative works on different terms, provided the original work is properly cited, appropriate credit is given, any changes made indicated, and the use is non-commercial. See: http://creativecommons.org/licenses/by-nc/4.0/.

ORCID iDs
Galia Jacobson http://orcid.org/0000-0002-1862-1468
Camilla Zimmermann http://orcid.org/0000-0003-4889-0244
Marcin Miszczyk http://orcid.org/0000-0002-4375-0827
Yaacov R. Lawrence http://orcid.org/0000-0002-9959-2485

REFERENCES

1 Kelsen DP, Portenoy RK, Thaler HT, *et al*. Pain and depression in patients with newly diagnosed pancreas cancer. *J Clin Oncol* 1995;13:748–55.
2 Lakatos G, Balázs A, Kui B, *et al*. Pancreatic cancer: multicenter prospective data collection and analysis by the Hungarian pancreatic Study Group. *J Gastrointestin Liver Dis* 2016;25:219–25.
3 Gencer D, Kästle-Larralde N, Pilz LR, *et al*. Presentation, treatment, and analysis of prognostic factors of terminally ill patients with gastrointestinal tumors. *Onkologie* 2009;32:380–6.
4 Grahm AL, Andrén-Sandberg A. Prospective evaluation of pain in exocrine pancreatic cancer. *Digestion* 1997;58:542–9.
5 Arthur AE, Delk A, Demark-Wahnefried W, *et al*. Pancreatic cancer survivors' preferences, barriers, and facilitators related to physical activity and diet interventions. *J Cancer Surviv* 2016;10:981–9.
6 Müller-Nordhorn J, Roll S, Böhmig M, *et al*. Health-Related quality of life in patients with pancreatic cancer. *Digestion* 2006;74:118–25.
7 Siegel RL, Miller KD, Jemal A. Cancer statistics, 2016. *CA Cancer J Clin* 2016;66:7–30.
8 Ferlay J, Colombet M, Soerjomataram I, *et al*. Cancer incidence and mortality patterns in Europe: estimates for 40 countries and 25 major cancers in 2018. *Eur J Cancer* 2018;103:356–87.
9 Wu W, He X, Yang L, *et al*. Rising trends in pancreatic cancer incidence and mortality in 2000-2014. *Clin Epidemiol* 2018;10:789–97.
10 Cherny NI. How to deal with difficult pain problems. *Ann Oncol* 2005;16 Suppl 2:ii79–87.
11 Lahoud MJ, Kourie HR, Antoun J, *et al*. Road map for pain management in pancreatic cancer: a review. *World J Gastrointest Oncol* 2016;8:599.
12 Brelin S, Fredheim OM, Loge JH, *et al*. Opioids for outpatients with cancer in their last year of life: a nationwide pharmacoepidemiological study. *J Opioid Manag* 2016;12:25–36.
13 Arcidiacono PG, Calori G, Carrara S, *et al*. Celiac plexus block for pancreatic cancer pain in adults. *Cochrane Database Syst Rev* 2011;3:CD007519.
14 Levy MJ, Wiersema MJ. EUS-guided celiac plexus neurolysis and celiac plexus block. *Gastrointest Endosc* 2003;57:923–30.
15 Zhu Z, Friess H, diMola FF, *et al*. Nerve growth factor expression correlates with perineural invasion and pain in human pancreatic cancer. *J Clin Oncol* 1999;17:2419.
16 Kambadakone A, Thabet A, Gervais DA, *et al*. Ct-Guided celiac plexus neurolysis: a review of anatomy, indications, technique, and tips for successful treatment. *Radiographics* 2011;31:1599–621.
17 Mercadante S. Celiac plexus block versus analgesics in pancreatic cancer pain. *Pain* 1993;52:187–92.
18 Wong GY, Schroeder DR, Carns PE, *et al*. Effect of neurolytic celiac plexus block on pain relief, quality of life, and survival in patients with unresectable pancreatic cancer: a randomized controlled trial. *JAMA* 2004;291:1092–9.
19 Kanno Y, Koshita S, Masu K, *et al*. Efficacy of EUS-guided celiac plexus neurolysis compared with medication alone for unresectable pancreatic cancer in the oxycodone/fentanyl era: a prospective randomized control study. *Gastrointest Endosc* 2020;92:120–30.
20 Burris HA, Moore MJ, Andersen J, *et al*. Improvements in survival and clinical benefit with gemcitabine as first-line therapy for patients with advanced pancreas cancer: a randomized trial. *J Clin Oncol* 1997;15:2403–13.
21 Conroy T, Desseigne F, Ychou M, *et al*. Folfirinox versus gemcitabine for metastatic pancreatic cancer. *N Engl J Med Overseas Ed* 2011;364:1817–25.
22 Beesley VL, Wockner LF, O'Rourke P, *et al*. Risk factors for current and future unmet supportive care needs of people with pancreatic cancer. A longitudinal study. *Support Care Cancer* 2016;24:3589–99.
23 Morganti AG, Trodella L, Valentini V, *et al*. Pain relief with short-term irradiation in locally advanced carcinoma of the pancreas. *J Palliat Care* 2003;19:258–62.
24 Wolny-Rokicka E, Sutkowski K, Grządziel A, *et al*. Tolerance and efficacy of palliative radiotherapy for advanced pancreatic cancer: a retrospective analysis of single-institutional experiences. *Mol Clin Oncol* 2016;4:1088–92.
25 Wong AA, Delclos ME, Wolff RA, *et al*. Radiation dose considerations in the palliative treatment of locally advanced adenocarcinoma of the pancreas. *Am J Clin Oncol* 2005;28:227–33.
26 Zimmermann FB, Jeremic B, Lersch C, *et al*. Dose escalation of concurrent hypofractionated radiotherapy and continuous infusion 5-FU-chemotherapy in advanced adenocarcinoma of the pancreas. *Hepatogastroenterology* 2005;52:246–50.
27 Lawrence YR, Hammer L, Morag O, *et al*. *Abstract CT147: celiac plexus radiosurgery a new palliative modality for upper gastrointestinal malignancies-final quality of life results from a proof-of-concept clinical trial*. AACR, 2019.
28 Bijur PE, Latimer CT, Gallagher EJ. Validation of a verbally administered numerical rating scale of acute pain for use in the emergency department. *Acad Emerg Med* 2003;10:390–2.
29 Paice JA, Cohen FL. Validity of a verbally administered numeric rating scale to measure cancer pain intensity. *Cancer Nurs* 1997;20:88–93.
30 Ahlers SJGM, van Gulik L, van der Veen AM, *et al*. Comparison of different pain scoring systems in critically ill patients in a general ICU. *Crit Care* 2008;12:R15–18.
31 Holdgate A, Asha S, Craig J, *et al*. Comparison of a verbal numeric rating scale with the visual analogue scale for the measurement of acute pain. *Emerg Med* 2003;15:441–6.
32 Feldman DB, Rand KL, Kahle-Wrobleski K. Hope and goal attainment: testing a basic prediction of hope theory. *J Soc Clin Psychol* 2009;28:479–97.
33 Pelayo-Alvarez M, Perez-Hoyos S, Agra-Varela Y. Reliability and concurrent validity of the palliative outcome scale, the Rotterdam symptom checklist, and the brief pain inventory. *J Palliat Med* 2013;16:867–74.
34 Harris K, Li K, Flynn C, *et al*. Worst, average or current pain in the brief pain inventory: which should be used to calculate the response to palliative radiotherapy in patients with bone metastases? *Clin Oncol* 2007;19:523–7.
35 Koedijk MS, Heijmen BJM, Groot Koerkamp B, *et al*. Protocol for the strong trial: stereotactic body radiation therapy following chemotherapy for unresectable perihilar cholangiocarcinoma, a phase I feasibility study. *BMJ Open* 2018;8:e020731.
36 Cleeland CS, Ryan K. *The brief pain inventory. pain Research Group*, 1991: 143–7.
37 Health UDo, Services H. *Common terminology criteria for adverse events (CTCAE) version 4.0*. 4. National Institutes of Health, National Cancer Institute, 2009.
